# METTL9 mediated N1-Histidine methylation of SLC39A7 confers ferroptosis resistance and inhibits adipogenic differentiation in mesenchymal stem cells

**DOI:** 10.1186/s10020-025-01271-w

**Published:** 2025-05-26

**Authors:** Jiahao Jin, Quanfeng Li, Yunhui Zhang, Pengfei Ji, Xinlang Wang, Yibin Zhang, Zihao Yuan, Jianan Jiang, Guangqi Tian, Mingxi Cai, Pei Feng, Yanfeng Wu, Peng Wang, Wenjie Liu

**Affiliations:** 1 Department of Orthopedics, The Eighth Affiliated Hospital of Sun Yat-Sen University, Shenzhen, P.R. China; 2 Guangdong Provincial Clinical Research Center for Orthopedic Diseases, The Eighth Affiliated Hospital, Sun Yat-sen University, Shenzhen, 518033 P.R. China; 3 Center for Biotherapy, The Eighth Affiliated Hospital of Sun Yat-Sen University, Shenzhen, P.R. China

**Keywords:** METTL9, SLC39A7, Mesenchymal stem cells, Adipogenic differentiation, Osteoporosis, Ferroptosis

## Abstract

Osteoporosis is a prevalent systemic metabolic disease, and an imbalance in the adipogenic and osteogenic differentiation of mesenchymal stem cells (MSCs) plays a crucial role in its pathogenesis. Thus, elucidating the mechanisms that regulate MSC lineage allocation is urgently needed. METTL9 was recently characterized as a novel N1-histidine methyltransferase that performs a wide range of functions. however, the role of METTL9 in the imbalance of MSC differentiation in osteoporosis remains unclear. In this study, we found that METTL9 expression was downregulated in osteoporosis, and further adipogenic functional experiments revealed that METTL9 negatively regulated the adipogenic differentiation of MSCs both in vitro and in vivo. Mechanistically, METTL9 mediated methylation of SLC39A7 at the His45 and His49 residues suppressed ferroptosis through the endoplasmic reticulum (ER) stress regulatory protein kinase R-like endoplasmic reticulum kinase (PERK)/ATF4 signaling pathway and the downstream protein SLC7A11. Moreover, SLC7A11 transported cystine for intracellular glutathione synthesis, eliminating intracellular reactive oxygen species (ROS) and inhibiting MSC adipogenic differentiation. Additionally, METTL9 overexpression significantly alleviated bone loss in ovariectomy (OVX) model mice. In summary, our results suggest that the METTL9/SLC39A7 axis may be a promising diagnostic and therapeutic target for osteoporosis.

**Supplementary Information:**

The online version contains supplementary material available at 10.1186/s10020-025-01271-w.

## Introduction

Osteoporosisis a systemic bone metabolic disease that is characterized by low bone mass and deterioration of bone microarchitecture, which lead to increased skeletal fragility and susceptibility to fracture (Compston et al. 2019). Osteoporosis occurs due to abnormalities in the bone remodeling process, including changes in the differentiation of multiple cell lineages. Bone marrow mesenchymal stem cells (MSCs) are multipotent stem cells that can differentiate into multiple cell types, such as chondrocytes, osteoblasts, and adipocytes (Pittenger et al. 1999). The multipotency of MSCs suggests the occurrence of competitive differentiation among different cell lineages. Typically, the reciprocal balance between MSC osteogenic and adipogenic differentiation is subject to tight spatiotemporal control to protect skeletal health, Disruption of this balance may result in osteoporosis (Kawai et al. 2009; Muruganandan et al. 2009; Rosen et al. 2006). Thus, maintaining a balance between the osteogenic and adipogenic differentiation of MSCs is essential for maintaining bone homeostasis, and limiting the adipogenic differentiation of MSCs can increase bone formation (Picke et al. 2018; Zhou et al. 2014). However, the specific mechanisms that affect MSC lineage allocation remain unclear.

Protein histidine methylation is a widespread posttranslational modification (PTM) in mammals that affects the functions of proteins by altering their structures and physicochemical properties (Kapell et al. 2021; Ning et al. 2016). Histidine can be methylated at either the N1 or N3 position of its imidazole ring, and several research groups have shown that SETD3 and METTL18 as N3 histidine methyltransferases in mammals (Guo et al. 2019; Kwiatkowski et al. 2018; Małecki et al. 2021; Wilkinson n.d.). The first N1 histidine methyltransferase, namely METTL9, was not reported until 2021 (Davydova et al. 2021; Lv 2021). METTL9 has a wide range of protein substrates and is involved in various cellular processes, such as promoting the migration and invasion of tumor cells, inhibiting the anti-Staphylococcus aureus activity of neutrophils, and reducing the activity of mitochondrial complex I (Davydova et al. 2021; Lv 2021; Cao 2023). However, despite the widespread presence of METTL9 mediated histidine methylation in mammals, its role in regulating MSC differentiation remains unknown.

SLC39A7 is an intracellular zinc transporter that is localized to the endoplasmic reticulum (ER) and is considered a critical “gatekeeper”, it regulates the release of zinc from the ER into the cytoplasm and play a vital role in maintaining zinc homeostasis in the ER (Woodruff et al. 2018). Dysfunction of SLC39A7 leads to the abnormal accumulation of zinc in the ER, inducing ER stress and triggering the unfolded protein response (UPR) (Bin et al. 2017). Previous studies have demonstrated that the inhibition of SLC39A7 function can enhance cellular resistance to ferroptosis by inducing ER stress (Chen et al. 2021). Ferroptosis is a new form of cell death distinct from apoptosis. Numerous studies have confirmed that ferroptosis plays a crucial role in the development of osteoporosis, and targeting ferroptosis is considered a promising direction for research on strategies to treat osteoporosis (Ru et al. 2023). However, previous investigations have focused primarily on the mechanisms that cause cells to undergo ferroptosis, with limited investigation on how ferroptosis affects MSC differentiation.

In this study, we aimed to reveal the biological role of METTL9 in the progression of osteoporosis. These results indicate that METTL9 regulates MSC differentiation by mediating the histidine methylation of SLC39A7, which in turn inhibits ferroptosis. Mechanistically, the METTL9 mediated methylation of SLC39A7 at the His45 and His49 residues impairs the zinc-binding activity of SLC39A7, leading to abnormal zinc accumulation in the ER. More importantly, abnormal zinc accumulation triggers ER stress, activates the protein kinase R-like endoplasmic reticulum kinase (PERK)/ATF4 pathway of the unfolded protein response, and upregulates the expression of the downstream protein SLC7A11. SLC7A11 is a critical inhibitor of ferroptosis, and it is responsible for transporting extracellular cystine for glutathione synthesis, thus suppressing intracellular oxidative stress and negatively regulating MSC adipogenic differentiation. These results indicate that the METTL9/SLC39A7 axis may be a potential diagnostic and therapeutic target for osteoporosis.

## Methods and materials

### Data sources

Gene expression profile data including the GSE35956 and GSE35958 datasets, which contain data from osteoporosis patients and healthy individuals, as well as the GSE83097 dataset, which contains RNA-seq data from SLC39A7-knockdown human mesenchymal stem cells (hMSCs), were downloaded from the GEO database. DEGs were analyzed via GEO-2R, with a LogFC threshold of less than − 1 or greater than 1 and an adjusted *P*-value of less than 0.05. KEGG pathway enrichment analysis and GSEA were conducted via R packages.

### Isolation and culture of human MSCs

Bone marrow was extracted from the posterior superior iliac spine of healthy volunteers under sterile conditions. The isolation and purification of bone marrow MSCs were performed by density gradient centrifugation. Briefly, the bone marrow samples were centrifuged at 2500 × g for 30 min to obtain purified MSCs. The isolated MSCs were then resuspended in 90% Dulbecco’s modified Eagle medium (DMEM; Gibco, 11885-076) supplemented with 10% fetal bovine serum (FBS; Thermo Fisher, 10099141), seeded into culture flasks, and incubated at 37 °C in 5% CO₂. The culture medium was replaced every three days to remove nonadherent cells. When the cells reached 80–90% confluence, they were trypsinized and passaged into new culture flasks or plates. Unless otherwise specified, all the experiments used MSCs from passages 2 to 5.

### Western blotting and immunoprecipitation assays

To extract proteins, the cells were lysed on ice for 30 min in RIPA buffer supplemented with 1% protease inhibitor and 1% phosphatase inhibitor. The lysates were then centrifuged at 14,000 rpm for 30 min at 4 °C, and the supernatants were collected. Protein concentrations were determined with a BCA protein assay kit (CWBIO, CW0014S). The protein samples were mixed with 5× sodium dodecyl sulfate (SDS) loading buffer at a 4:1 ratio and heated. For immunoprecipitation, the supernatants of the cell lysates were precleared with protein A/G magnetic beads (Invitrogen, #88802) and then incubated overnight at 4 °C with the designated antibody or control IgG. After incubation, magnetic beads were added to the samples, which were subsequently incubated for 3 h at 4 °C. The immunoprecipitates were washed, mixed with sample loading buffer, and heated. Proteins were separated by SDS‒polyacrylamide gel electrophoresis (SDS‒PAGE) and then transferred to polyvinylidene difluoride (PVDF) membranes (Millipore, IPVH0010). The membranes were blocked in 5% nonfat milk for 1 h and incubated overnight with primary antibodies at 4 °C. The membranes were washed three times with Tris-buffered saline with Tween (TBST) to eliminate nonspecific binding and then incubated for 1 h at room temperature with HRP-conjugated secondary antibodies (1:3,000, Boster, BA1050 & BA1054). Finally, the membranes were washed with TBST and visualized with Immobilon Western Chemiluminescent HRP Substrate (Millipore WBKLS0500). Primary antibodies used in this study are as follows: METTL9 (Proteintech, 15120-1-AP), SLC39A7 (Proteintech, 19429-1-AP), PPAR-γ (Abcam, ab178860), C/EBPα (Abcam, ab40764), FABP4 (Abcam, ab92501), GRP78 (Proteintech, 66574-1-Ig), PERK (Proteintech, 24390-1-AP), p-PERK (Proteintech, 29546-1-AP), ATF4 (Proteintech, 108351-AP), SLC7A11 (Proteintech, 26864-1-AP), pan-methyl-Histidine (ABclonal, A17948), Flag (CST, 14793 S), GAPDH (Cell Signaling Technology, 5174 S), 𝛽-ACTIN (Abcam, ab8226).

### RNA extraction and RT‒qPCR

According to the manufacturer’s protocol, total RNA was isolated from MSCs with Trizol solution (Invitrogen, #15596-026), and RNA was reverse-transcribed into complementary DNA with Evo M-MLV RT Premix (AG, #11706). RT‒qPCR was performed using the SYBR Green Premix Pro Taq HS qPCR Kit (AG, #11718). Gene expression levels were quantified with a real-time quantitative PCR system (Applied Biosystems, 7500), and GAPDH was used as the normalization control. The 2 − ΔΔCt method was used to calculate relative expression levels. The sequences of the primer that were used for target gene analysis in this study are listed in Supplementary file 1.

### RNA interference

siRNAs targeting METTL9, SLC39A7, CCNT2, DNAJB12, and the negative control were designed and produced by GenePharma (Suzhou, China). Three different siRNAs were designed for each gene, and the most effective siRNA was used for the experiments. When cells reached 70–90% confluence, transfection was performed with Lipofectamine RNAiMAX (Thermo Fisher, 13778150) according to the manufacturer’s instructions. The transfection efficiency was evaluated 48 h post-transfection by qPCR or 72 h post-transfection by Western blotting.

### Lentivirus, and adenovirus generation and infection

The vector control, wild-type SLC39A7, and mutant SLC39A7 overexpression lentiviruses, along with the METTL9 overexpression lentivirus and adenovirus, were designed and synthesized by OBiO Technology (Shanghai). Once cells reached confluence 60–70%, transduction was performed. The virus volume was calculated on the basis of the viral titer and cell number (12-well plate: 0.6 × 10⁵ cells/well). Lentivirus (MOI = 30), polybrene (5 µg/ml), and complete DMEM were thoroughly mixed and added to the cell culture plates. The plates were incubated at 37 °C, and the viral infection medium was replaced after 24 h. The transduction efficiency was analyzed 3 days post-transduction for further experiments.

### Adipogenic differentiation assay in vivo

To induce in vivo adipogenic differentiation, MSCs that had been transducted with lentivirus or siRNA were cultured in adipogenic differentiation medium, and the experiment was conducted after 4–5 days of adipogenic differentiation. The night before the experiment, Matrigel (Corning, Cat. No. 356237) was thawed at 4 °C. On the day of the experiment, pipette tips and EP tubes were precooled. MSCs were digested with trypsin and collected into 1.5 ml EP tubes. For every 1.5 × 10⁴ MSCs, 100 µl with cold, serum-free high-glucose DMEM was added, followed by the addition of an equal volume of Matrigel. The mixture was gently pipetted to obtain the final Matrigel-cell suspension. After each nude mouse was anesthetized, 200 µl of the Matrigel-cell suspension was subcutaneously injected. The Matrigel was retrieved for HE staining and immunohistochemical analysis 6–8 weeks later.

### H&E and Masson staining

Femurs were collected from mice and fixed in 4% paraformaldehyde for 1–2 days, followed by decalcification using EDTA decalcifying solution (BOSTER, AR1071) until a fine needle could easily penetrate the bone. The decalcified samples were embedded in paraffin and sectioned into 5 μm thick slices for H&E and Masson staining. The sections were first deparaffinized in xylene, rehydrated in graded ethanol solutions, and washed with distilled water. For H&E staining, hematoxylin solution (Servicebio, #G1004) was added to the sections, which were then incubated for 8 min, followed by washing with running tap water for 10 min. The sections were then differentiated with 1% hydrochloric acid alcohol for 5 s, washed with PBS, and stained with eosin for 8 min. After staining, the sections were washed, dehydrated, cleared, and mounted with neutral resin. The stained sections were observed and photographed under a light microscope. For Masson staining, a Masson trichrome staining kit (Sigma, USA) was used according to the manufacturer’s instructions. The sections were then washed with PBS, dehydrated, cleared, and mounted with neutral resin. The stained sections were observed and photographed under a light microscope.

### Immunohistochemistry and tissue immunofluorescence staining

As described above, femur sections from mice were obtained. The sections were deparaffinized, rehydrated, and placed in a beaker containing sodium citrate buffer for antigen retrieval by heating in a 60 °C water bath for 12 h. After the sections were cooled to room temperature, they were incubated with 3% hydrogen peroxide for 20 min, washed with PBS, and then blocked with 10% goat serum for 30 min. Next, diluted primary antibodies were added, and the sections were incubated overnight at 4 °C. The following day, secondary antibody incubation and color development were performed with a mouse HRP kit (DAB, CW2069S) according to the manufacturer’s instructions. For immunofluorescence staining, 0.1% Triton X-100 was used to permeabilize the cells or tissue sections for 15 min, followed by three washes with PBS. The sections were then blocked with 10% goat serum for 30 min and incubated with diluted primary antibodies overnight at 4 °C. After being washed with PBS, the sections were incubated with species-matched fluorescently labeled secondary antibodies in the dark at room temperature for 1 h. Finally, the stained sections were dehydrated and mounted, and the results were observed and photographed by fluorescence or confocal microscopy.

### Adipogenic differentiation of hMSCs in vitro and ORO staining

To induce in vitro adipogenic differentiation, we replaced the regular medium with adipogenic differentiation medium when cells reached 80–90% confluence. The adipogenic differentiation induction medium consisted of high-glucose DMEM supplemented with 10% FBS, 1 µM dexamethasone, 10 µg/ml insulin, 0.5 mM IBMX, 0.2 mM indomethacin, and 100 IU/ml penicillin-streptomycin. The adipogenic induction medium was freshly prepared and changed every three days. After MSCs were induced to undergo adipogenic differentiation for two weeks, the medium was discarded, and the cells were fixed with 4% paraformaldehyde for 25 min. After being washed three times with PBS, the cells were stained at room temperature with ORO staining solution (Beyotime, C0157S) for 15 min. The stained cells were then observed under a microscope and photographed. To quantify the ORO staining results, isopropanol was added to the wells for 60 min to extract the dye, and the absorbance was measured at a wavelength of 510 nm. The values were recorded, and each group was quantitatively analyzed.

### Cellular ROS detection

To measure the intracellular ROS levels, the cells were stained with 10 µM DCFH-DA (Beyotime, S0033) in an incubator in the dark at 37 °C for 20 min. The cells were then washed with serum-free medium, DCF fluorescence intensity was measured by flow cytometry (BD Bioscience, BD Influx), and the results were analyzed with FlowJo V10 software.

### Mitochondrial ROS measurement

Mitochondrial ROS levels were measured with the MitoSOX™ Red Mitochondrial Superoxide Indicator (Thermo Fisher, M36008). The MitoSOX probe was diluted to a working concentration of 5 µM and incubated with the cells for 15 min at 37 °C in the dark. After washing with PBS, the cell nuclei were stained with DAPI. The fluorescence intensity of MitoSOX was quantified by ImageJ software.

### Lipid peroxidation assay

Lipid peroxidation levels were detected with a C11-BODIPY probe (Invitrogen, D3861). The C11-BODIPY probe was diluted to a working concentration of 10 µM and incubated with the cells at 37 °C for 30 min. After washing with PBS, the cells were collected and analyzed via flow cytometry.

### GSH and cysteine assays

The total intracellular GSH and cysteine levels were measured with the GSH Assay Kit (Solarbio, Beijing, China) and the Cysteine Assay Kit (Solarbio, Beijing, China) according to the manufacturers’ instructions. The total protein levels in each group of cells were then measured with a BCA protein quantification kit (CWBIO, CW0014S), and the GSH and Cys levels were normalized to the total protein concentration and analyzed.

### Establishment and treatment of the mouse osteoporosis model

C57BL/6J mice aged 6–8 weeks were obtained from the Sun Yat-sen University Experimental Animal Center. When the mice reached 16 months of age, they were used as a model of senile osteoporosis. To establish the postmenopausal osteoporosis mouse model, 8-week-old female wild-type C57BL/6 mice underwent bilateral ovariectomy or sham surgery. One week after the osteoporosis mouse model was established, the mice were injected via the tail vein with either control adenovirus or METTL9-overexpressing adenovirus (1 × 10^11 vg/g). Twelve weeks later, the femurs of the mice were collected for micro-CT analysis, H&E staining, Masson staining, and immunohistochemical and immunofluorescence staining.

### Micro‑CT

To assess femoral bone mass, the femur samples to be tested were fixed in 4% paraformaldehyde for 24 h, and then, the entire length was scanned by small animal micro-CT. Micro-CT analysis software was used to measure morphological parameters and perform 3D reconstruction of the region 50 mm distal to the growth plate, including 100 scan layers at the distal femur. Trabecular parameters including bone volume/total volume (BV/TV), bone surface area/bone volume (BSA/BV), trabecular thickness (Tb.Th), trabecular number (Tb.N), trabecular spacing (Tb.Sp), and cortical wall thickness (Ct.Th) were recorded.

### Statistical analysis

The data were statistically analyzed with GraphPad Prism 8 and SPSS 22.0 software, and all the results are presented as the means ± standard deviations (SDs). Significant differences between two groups were analyzed via Student’s t-test, and for comparisons among more than two groups, one-way analysis of variance (ANOVA) was used. Pearson correlation coefficients were calculated for correlation analysis. A *P*-value of less than 0.05 was considered statistically significant.

## Results

### METTL9 expression is downregulated in individuals with osteoporosis

**Fig. 1:**
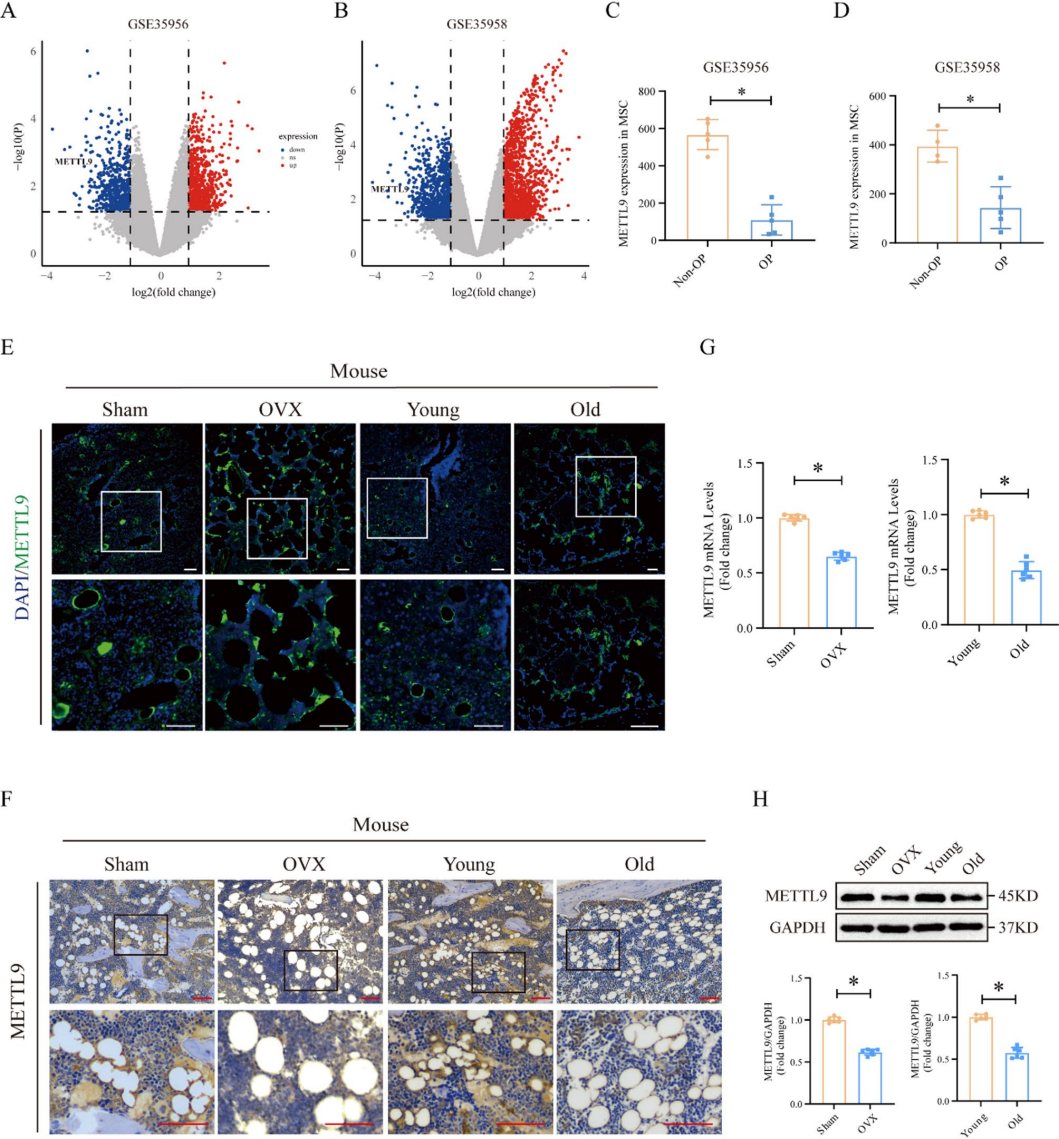
METTL9 expression is downregulated in individuals with osteoporosis. **A**, **B** Volcano plots showing that METTL9 expression was significantly downregulated in both datasets. **C**, **D** The expression of METTL9 in the GEO dataset GSE35956 and GSE35958 was analyzed. **E** Immunofluorescence and (**F**) immunohistochemical staining showing METTL9 expression in the femurs of sham-operated, OVX-treated, young, and aged C57BL/6J mice. **G** qRT–PCR and (**H**) Western blotting were performed to measure the METTL9 mRNA and protein levels in the bone marrow mesenchymal stem cells of sham-operated, OVX-treated, young, and aged C57BL/6J mice. The scale bar in the images represents 100 μm (10×). Data are presented as mean ± SD of six biologically independent replicates, *p < 0.05

To elucidate the potential role of METTL9 in osteoporosis, we initially used data from the Gene Expression Omnibus (GEO) database to analyze METTL9 expression levels in osteoporosis patients, specifically, we obtained two gene expression datasets (GSE35956 and GSE35958) that contain data from primary osteoporosis patients and healthy controls. Differentially expressed genes (DEGs) were identified in the two datasets, as shown by the Volcano plots (Fig. 1A, B) that METTL9 expression was downregulated in both datasets. We then conducted differential gene expression analysis of these two datasets. Surprisingly, we found that METTL9 expression in MSCs from OP patients was significantly downregulated (Fig. 1C, D).

To further confirm the results of GEO data analysis, we subjected 8-week-old C57BL/6J mice to ovariectomy (OVX) to establish a model of postmenopausal osteoporosis, and mice that were subjected to a sham-operation mice served as controls. Eighteen-month-old C57BL/6J mice were used to model osteoporosis in an elderly population, while 8-week-old C57BL/6J mice served as young controls. Through immunohistochemical and immunofluorescence staining, we found that METTL9 expression in the bone tissues of OVX-treated and aged osteoporotic mice was significantly lower than that in the bone tissues of the sham-operated and young groups (Fig. 1E, F). We then isolated MSCs from the femoral bone marrow of osteoporotic and control mice and further assessed METTL9 expression in the MSCs. The results revealed that the METTL9 mRNA and protein levels were significantly downregulated in osteoporotic mice (Fig. 1G, H), which is consistent with the bone tissue section results.

In summary, we confirmed that METTL9 expression was significantly downregulated in the osteoporosis group at the bioinformatics, tissue, and cellular levels. Thus, we hypothesize that the progression of osteoporosis is closely associated with the downregulation of METTL9 expression. 

### METTL9 negatively regulates the adipogenic differentiation of MSCs in vitro and in vivo

**Fig. 2:**
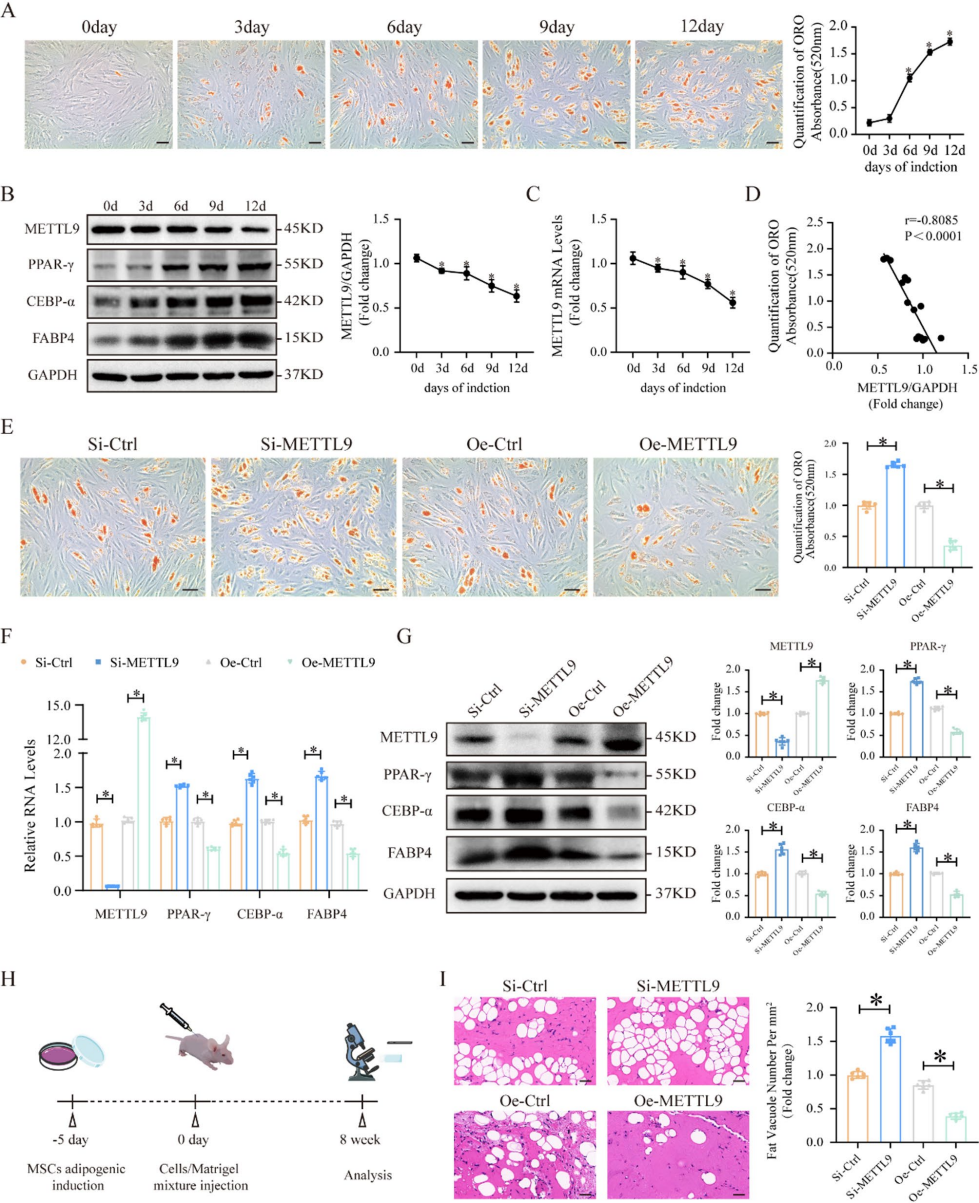
Decreasing METTL9 expression promotes the adipogenic differentiation of MSCs in vitro and in vivo. **A** ORO staining and quantification of MSCs at different time points during adipogenic differentiation. **B** Western blotting and **C** qRT–PCR analysis of changes in METTL9 protein and mRNA expression at various time points of adipogenic differentiation. **D** Correlation analysis between METTL9 expression levels and ORO staining during MSC adipogenic differentiation. **E** ORO staining and quantification of the adipogenic differentiation of METTL9-knockdown and METTL9-overexpression MSCs. **F** qRT–PCR and **G** Western blotting analysis of the adipogenic markers PPAR-γ, C/EBP-α, and FABP4 in METTL9-knockdown and METTL9-overexpression MSCs. **H** Schematic diagram of the in vivo culture experiment of MSCs in nude mice. **I **H&E staining showing the in vivo adipogenic differentiation and quantification in the METTL9-knockdown and METTL9-overexpressing groups. The scale bar in the images represents 50 μm. Data are presented as mean ± SD of six biologically independent replicates, *p < 0.05

The lineage differentiation of MSCs toward adipocytes results in progressive bone loss and increased bone marrow adipose tissue formation, thereby promoting the development of osteoporosis. Therefore, we conducted an in-depth investigation of the role of METTL9 in the adipogenic differentiation of MSCs. We first cultured MSCs in adipogenic differentiation medium (AD) for up to 12 days, and Oil Red O (ORO) staining revealed that the adipogenic differentiation of MSCs progressively increased from day 0 to day 12 (Fig. 2A). Moreover, Western blotting and quantitative real-time PCR (qRT–PCR) results indicated that METTL9 expression gradually decreased during adipogenic differentiation (Fig. 2B and C). Additionally, we analyzed the correlation between METTL9 expression levels and ORO staining and found that METTL9 expression was significantly negatively correlated with MSC adipogenic differentiation (Fig. 2D).

To further confirm the effect of METTL9 on the adipogenic differentiation of MSCs, we generated METTL9-knockdown siRNA and METTL9-overexpressing lentivirus, and either downregulated or overexpressed METTL9 in MSCs. After 7 days of adipogenic differentiation, ORO staining revealed that METTL9 downregulation significantly promoted MSC adipogenic differentiation, whereas METTL9 overexpression significantly inhibited this process (Fig. 2E). Moreover, we examined the expression of the adipogenic markers PPAR-γ, CEBP-α, and FABP4. Both the qRT–PCR and Western blotting results revealed that METTL9 downregulation significantly increased the expression of adipogenic markers in MSCs during adipogenic differentiation, whereas METTL9 overexpression significantly reduced the expression of these markers (Fig. 2F and G).

Additionally, we performed a mature human stem cell culture experiment in vivo to further assess the effect of METTL9 on the adipogenic differentiation of MSCs in vivo. After METTL9 was knocked down or overexpressed in MSCs, the cells were mixed with Matrigel and subcutaneously implanted into nude mice. After 6–8 weeks, the subcutaneous implants were removed and subjected to HE staining (Fig. 2H). The results revealed that METTL9 knockdown significantly increased the number of adipocytes, whereas METTL9 overexpression significantly reduced the number of adipocytes (Fig. 2I), which was consistent with the in vitro findings. In conclusion, our data suggest that METTL9 negatively regulates the adipogenic differentiation of MSCs both in vivo and in vitro.

### Zinc transporter SLC39A7 is involved in the METTL9 mediated regulation of MSC adipogenic differentiation

**Fig. 3:**
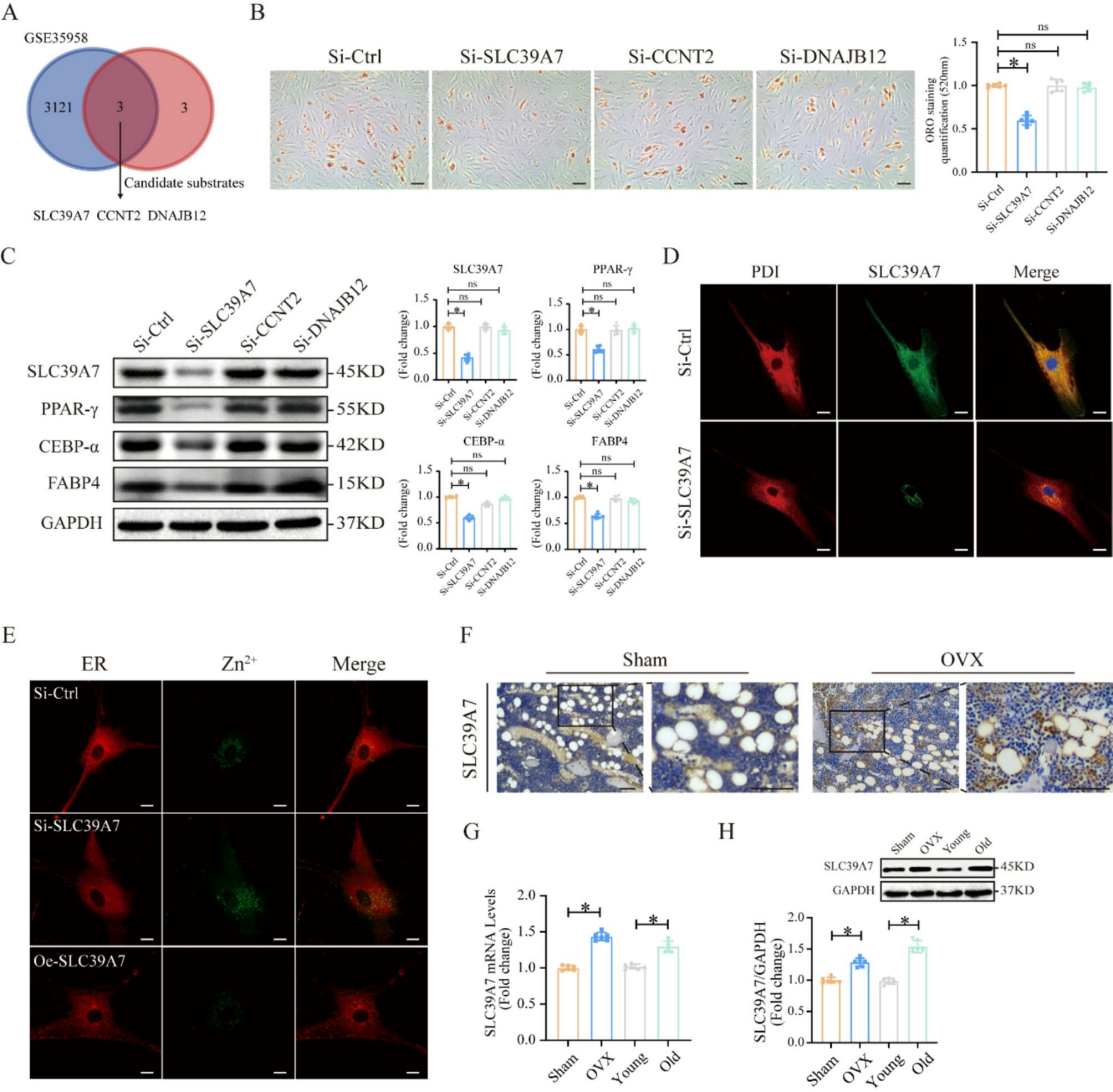
The Zinc Transporter SLC39A7 is involved in the METTL9 mediated regulation of MSC adipogenic differentiation. **A** Venn diagram showing three candidate genes. **B** ORO staining and quantification of MSC adipogenic differentiation after siRNA treatment. **C** Western blotting detection and quantification of the protein levels of the adipogenic markers PPAR-γ, C/EBP-α, and FABP4. **D** Confocal microscopy showing the colocalization of SLC39A7 with the ER. **E** Confocal microscopy was used to detect zinc levels within the ER. **F** Immunohistochemistry showing SLC39A7 expression in the femurs of sham-operated mice and OVX-treated mice. **G** qRT‒PCR and **H** Western blotting measurement of SLC39A7 mRNA and protein levels in MSCs from sham-operated mice, OVX-treated mice, young mice, and aged mice. **B** scale bar = 50um. **D**, **E** scale bar = 20um. **F** scale bar = 100um. Data are presented as mean ± SD of six biologically independent replicates, ns denotes no statistical significance, *p < 0.05

METTL9 has a wide range of substrates and can mediate the N1 histidine methylation of various proteins (Davydova et al. 2021). To further explore the mechanism by which METTL9 participates in MSC adipogenic differentiation, we compared the potential substrates of METTL9 with the DEGs from the GSE35958 (osteoporosis) dataset. As shown by the Venn diagram (Fig. 3A), we identified three candidate genes: SLC39A7, CCNT2, and DNAJB12. We performed adipogenic functional validation of these genes, after SLC39A7 knockdown, ORO staining revealed a significant decrease in adipocyte formation during MSC adipogenic differentiation (Fig. 3B). The Western blotting results revealed that the protein expression levels of adipogenic markers in MSCs were significantly decreased (Fig. 3C). In contrast, knockdown of CCNT2 and DNAJB12 had little effect on adipocyte formation during MSC adipogenic differentiation (Fig. 3B), and the protein expression levels of adipogenic markers in MSCs showed almost no change (Fig. 3C). Therefore, we selected SLC39A7 for further studies.

First, we demonstrated through immunofluorescence colocalization that SLC39A7 is localized to the ER in MSCs (Fig. 3D), We also found that mesenchymal stem cells lacking SLC39A7 accumulated zinc in the ER, while overexpressing SLC39A7 significantly reduced zinc in the ER (Fig. 3E). We then compared the expression levels of SLC39A7 in bone tissue sections from the sham-operated group and the OVX group, via immunohistochemistry. We found that SLC39A7 expression was significantly upregulated in the OVX-treated osteoporosis animal models (Fig. 3F). Further investigation of the SLC39A7 expression levels in MSCs revealed that, compared with the control group, the osteoporosis group had significantly elevated SLC39A7 mRNA and protein levels (Fig. 3G and H), which was consistent with the findings in bone tissue sections. In summary, these results suggest that SLC39A7 may be a key substrate through which METTL9 regulates the adipogenic differentiation of MSCs.

### METTL9 regulates the adipogenic differentiation of MSCs through the methylation of SLC39A7 at His45 and His49

**Fig. 4:**
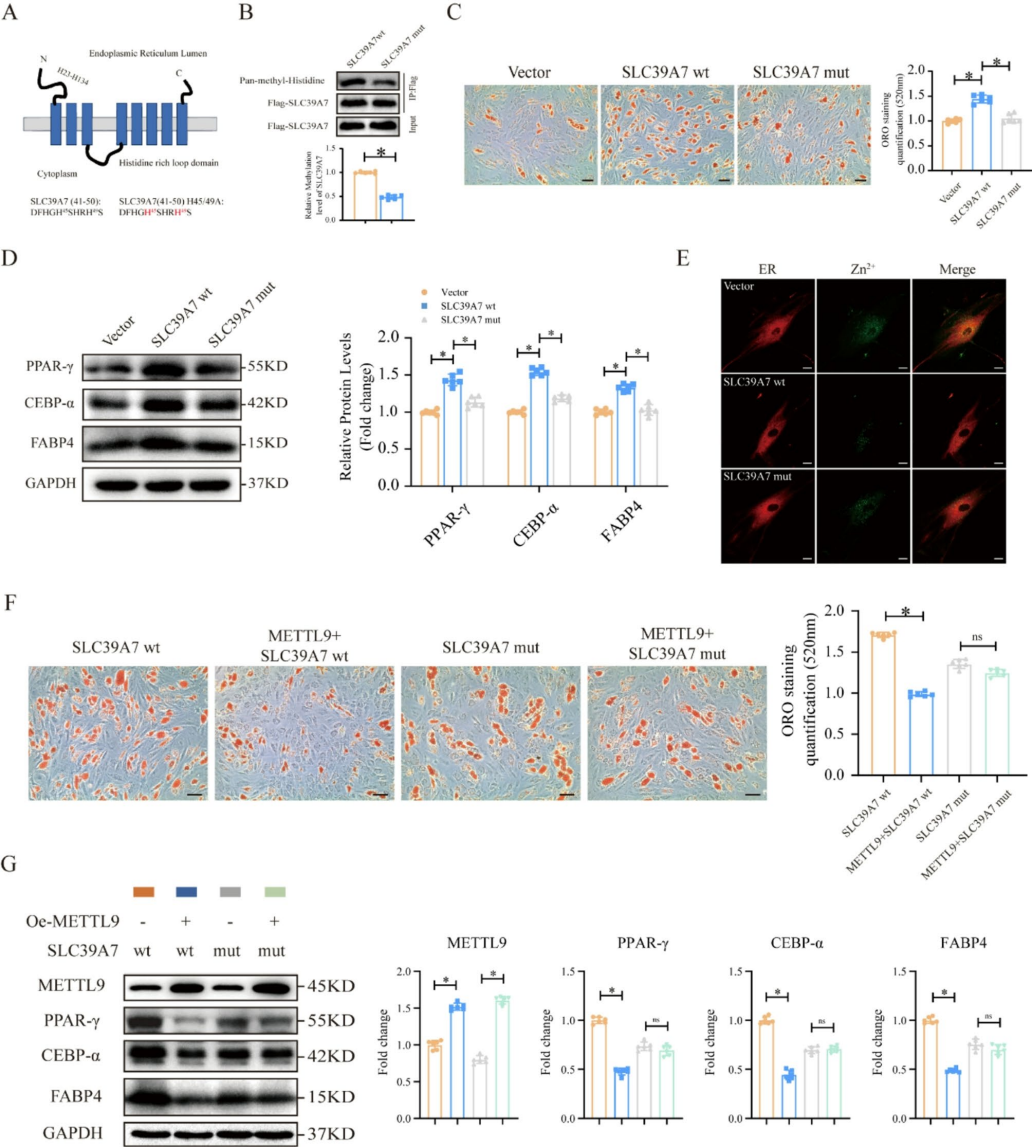
METTL9 regulates the adipogenic differentiation of MSCs through the methylation of SLC39A7 at His45 and His49. **A** Schematic diagram of SLC39A7 mutant generation. **B** Immunoblotting was used to measure the methylation levels of SLC39A7 wt and SLC39A7 mut. **C** ORO staining and quantification of the adipogenic differentiation of MSCs stably expressing Vector, SLC39A7 wt, or SLC39A7 mut. **D** Western blotting detection and quantification of adipogenic marker protein levels in MSCs stably expressing Vector, SLC39A7 wt, or SLC39A7 mut. **E** Confocal microscopy was used to detect zinc levels within the ER. **F** ORO staining and quantification of the adipogenic differentiation of MSCs stably expressing SLC39A7 wt + Vector, SLC39A7 wt + METTL9, SLC39A7 mut, or SLC39A7 mut + METTL9. **G** Western blotting detection and quantification of adipogenic marker protein levels in MSCs stably expressing SLC39A7 wt + Vector, SLC39A7 wt + METTL9, SLC39A7 mut, or SLC39A7 mut + METTL9. **C**, **F** scale bar = 50um. **E** scale bar = 20um. Data are presented as mean ± SD of six biologically independent replicates, ns denotes no statistical significance, *p < 0.05

According to previous studies, the N-terminal region of SLC39A7 is rich in highly conserved histidine residues, and the histidine residues at positions 45 and 49 have been identified as sites that are methylated by METTL9 (Davydova et al. 2021; Adulcikas et al. 2018). Therefore, to further explore the detailed mechanism underlying the histidine methylation of SLC39A7 during MSC adipogenic differentiation, we replaced the histidine residues at positions 45 and 49 with alanine, thus generating a SLC39A7 mutant (Fig. 4A). First, we confirmed that SLC39A7 could be methylated by METTL9 (Figure. 4-figure Supplement 1), whereas the histidine-to-alanine mutation suppressed the overall methylation of SLC39A7 (Fig. 4B). Compared with the wild-type SLC39A7 (SCL39A7 wt), ORO staining revealed that the SLC39A7 mutant significantly reduced the proportion of adipocytes that were generated during MSC adipogenic differentiation (Fig. 4C). The Western blotting results revealed that the protein levels of MSC adipogenic markers were significantly reduced (Fig. 4D). Consistent with these observations, the SLC39A7 mutant significantly impaired the ability of SLC39A7 wt to transport zinc, resulting in a marked accumulation of zinc within the ER (Fig. 4E). In summary, the methylation of the histidine residues at positions 45 and 49 of the SLC39A7 protein has a significant effect on the function of this protein.

Next, we investigated the effects of histidine methylation at the His45 and His49 sites of SLC39A7 on the METTL9 mediated regulation of MSC adipogenic differentiation. We designed four experimental combinations: SLC39A7 wt + vector, SLC39A7 wt + METTL9, SLC39A7 mut, and SLC39A7 mut + METTL9. We then assessed the adipogenic differentiation of MSCs and the expression of adipogenic markers by ORO staining and Western blotting. METTL9 overexpression significantly reduced the adipogenic differentiation of MSCs (Fig. 4F) and expression of adipogenic marker protein (Fig. 4G). However, this effect was not observed in MSCs expressing the SLC39A7 mutant (Fig. 4F and G). In summary, our results strongly suggest that histidine methylation at the His45 and His49 sites of SLC39A7 is crucial for METTL9 mediated MSC adipogenic differentiation.

### METTL9-SLC39A7 axis affects the adipogenic differentiation of MSCs by regulating ferroptosis

**Fig. 5:**
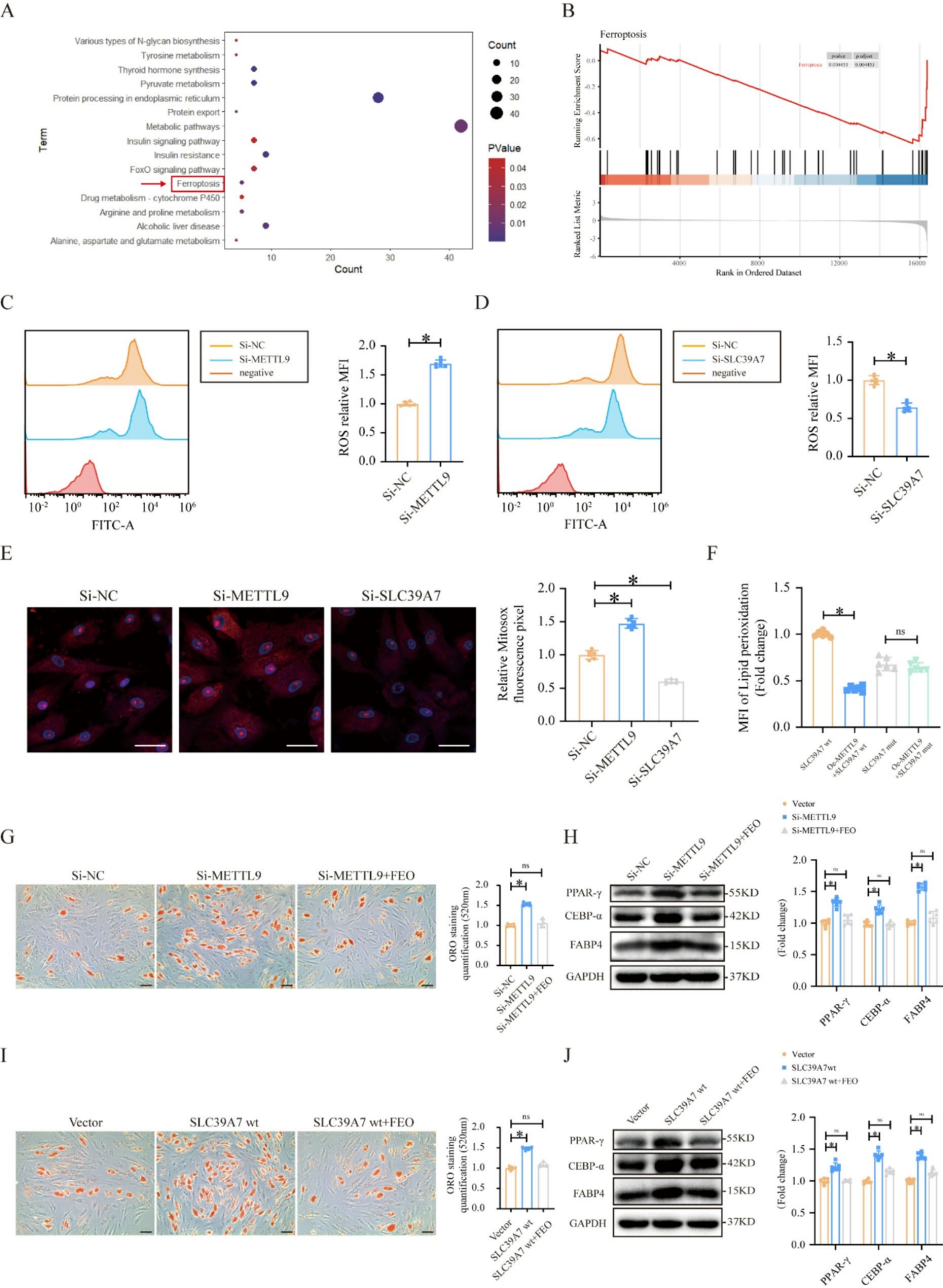
The METTL9‒SLC39A7 axis affects the adipogenic differentiation of MSCs by regulating ferroptosis. **A** KEGG pathway enrichment analysis of the GSE83097 dataset. **B** GSEA enrichment analysis of the GSE83097 dataset. **C** Total intracellular ROS levels after METTL9 knockdown. **D** Total intracellular ROS levels after SLC39A7 knockdown. **E** Mitosox signal intensity and quantification after METTL9 knockdown. **F** Flow cytometry anlysis and quantification of the mean fluorescence intensity (MFI) of lipid peroxidation in MSCs stably expressing SLC39A7 wt + Vector, SLC39A7 wt + METTL9, SLC39A7 mut, and SLC39A7 mut + METTL9. **G** ORO staining and quantification of MSC adipogenic differentiation in the si-NC, si-METTL9, and si-METTL9 + FEO groups. **H** Western blotting analysis and quantification of adipogenic marker protein levels in the si-NC, si-METTL9, and si-METTL9 + FEO groups. **I** ORO staining and quantification analysis of MSC adipogenic differentiation in the Vector, SLC39A7 wt, and SLC39A7 wt + FEO groups. **J** Western blotting analysis and quantification of adipogenic marker protein levels in the Vector, SLC39A7 wt, and SLC39A7 wt + FEO groups. The scale bar in the images represents 50 um. Data are presented as mean ± SD of six biologically independent replicates, ns denotes no statistical significance, *p < 0.05

To understand how METTL9 influences MSC adipogenic differentiation via the N1 histidine methylation of SLC39A7, we first downloaded the gene expression dataset GSE83097 from the GEO database, which includes data from SLC39A7-knockdown MSCs. Using GEO2R, we identified DEGs between the SLC39A7-KD and NC groups, and the DEGs were subjected to KEGG pathway enrichment analysis, the results revealed significant changes in the ferroptosis pathway (Fig. 5A). GSEA further confirmed the close association between SLC39A7 and the ferroptosis signaling pathway (Fig. 5B). Therefore, we hypothesized that the METTL9‒SLC39A7 axis influences MSC adipogenic differentiation by affecting the ferroptosis pathway.

To test this hypothesis, we first measured the levels of reactive oxygen species (ROS) and lipid peroxidation, which are closely related to ferroptosis, via flow cytometry. The results revealed that METTL9 knockdown significantly increased the total ROS levels in MSCs (Fig. 5C), whereas SLC39A7 knockdown significantly reduced the total ROS levels in MSCs (Fig. 5D). We also measured the mitochondrial ROS levels, and the results were consistent with the results for total intracellular ROS (Fig. 5E). Next, we designed four experimental groups: SLC39A7 wt + Vector, SLC39A7 wt + METTL9, SLC39A7 mut, and SLC39A7 mut + METTL9, and used flow cytometry to measure lipid peroxidation. The results showed that METTL9 overexpression significantly reduced the mean fluorescence intensity (MFI) of lipid peroxidation, but this effect was not observed in MSCs expressing the SLC39A7 mutant (Fig. 5F). These results suggest that METTL9 regulates ferroptosis through the methylation of SLC39A7.

Next, to determine whether METTL9 and SLC39A7 influence MSC adipogenic differentiation through ferroptosis, we performed ORO staining to measure MSC adipogenic differentiation and Western blotting to examine adipogenic marker expression. The results revealed that the ferroptosis inhibitor (FEO) suppressed the si-METTL9 mediated increase in MSC adipogenic differentiation and elevation in adipogenic marker protein levels (Fig. 5G and H). Similarly, it also suppressed the corresponding effects of SLC39A7 wt overexpression (Fig. 5I and J). In summary, these findings suggest that the METTL9‒SLC39A7 axis influences MSC adipogenic differentiation by regulating the ferroptosis pathway.

### METTL9-SLC39A7 axis regulates MSC ferroptosis through the ATF4/SLC7A11 pathway

**Fig. 6:**
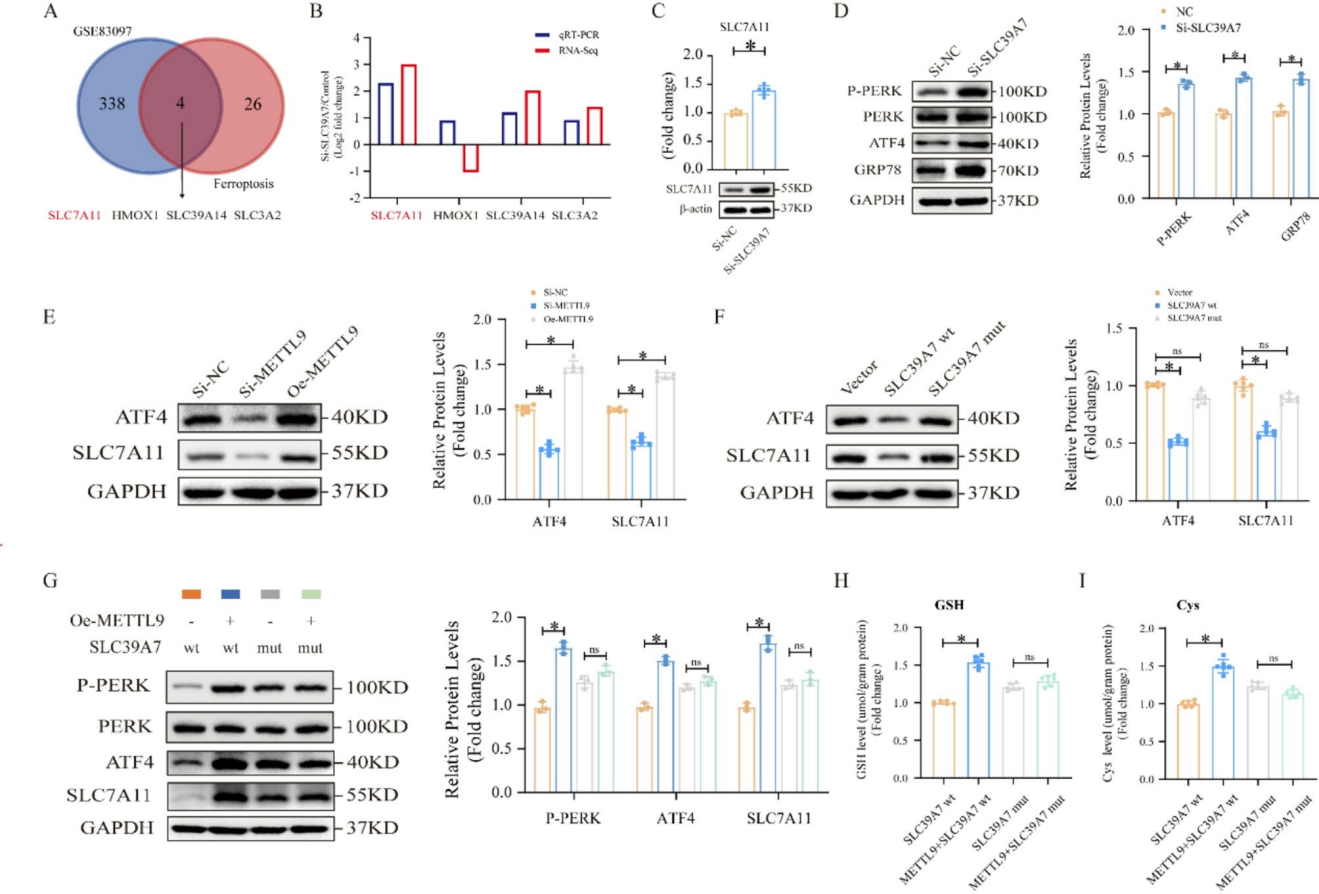
METTL9-SLC39A7 axis regulates MSC ferroptosis through the ATF4/SLC7A11 pathway. **A** Venn diagram identifying four ferroptosis-related candidate genes. **B** RT‒qPCR analysis of the mRNA expression of these ferroptosis-related genes after si-SLC39A7 treatment. **C** Western blotting analysis and quantification of SLC7A11 levels after si-SLC39A7 treatment. **D** Western blotting analysis and quantification of changes in the PERK/ATF4 pathway after si-SLC39A7 treatment. **E** Western blotting analysis and quantification of ATF4/SLC7A11 expression after METTL9 knockdown and overexpression. **F** Western blotting analysis and quantification of ATF4/SLC7A11 expression after stable expression of SLC39A7 wt and SLC39A7 mut. **G** Western blotting analysis and quantification of changes in the PERK/ATF4 pathway and SLC7A11 expression in MSCs stably expressing SLC39A7 wt + Vector, SLC39A7 wt + METTL9, SLC39A7 mut, and SLC39A7 mut + METTL9. **H**, **I** Intracellular GSH and CYS levels in MSCs stably expressing SLC39A7 wt + Vector, SLC39A7 wt + METTL9, SLC39A7 mut, and SLC39A7 mut + METTL9. The scale bar in the images represents 20 μm. Data are presented as mean ± SD of six biologically independent replicates, ns denotes no statistical significance, *p < 0.05

To explore how METTL9-SLC39A7 regulates ferroptosis, we compared the DEGs in the GSE83097 dataset with ferroptosis regulatory genes (Tang et al. 2022), and we identified four candidate genes: SLC7A11, HMOX1, SLC39A14, and SLC3A2 (Fig. 6A). Both the RNA-Seq and qRT‒PCR results indicated that SLC7A11 expression exhibited the most significant changes after SLC39A7 knockdown (Fig. 6B), and the protein levels of SLC7A11 were also significantly increased after si-SLC39A7 treatment (Fig. 6C).

Previous studies have shown that the loss of SLC39A7 function leads to ER stress and activates the UPR (Woodruff et al. 2018). To investigate this, we first used qRT‒PCR to assess changes in the three UPR signaling pathways after si-SLC39A7 treatment. The results revealed a significant increase in PERK expression, whereas there was no difference in inositol-requiring enzyme-1α (IRE-1α) or activating transcription factor 6 (ATF6) expression between the experimental and control groups (Figure. 6-figure supplement 1). We then used Western blotting to further assess the activation of the PERK/ATF4 signaling pathway and the expression levels of GRP78, which is a key chaperone protein in the ER stress response. SLC39A7 knockdown increased GRP78 expression and promoted PERK phosphorylation and the expression of its major effector ATF4 (Fig. 6D). ATF4 is the transcription factor for SLC7A11, and ATF4 expression is positively correlated with SLC7A11 expression. We hypothesize that the METTL9‒SLC39A7 axis may influence ferroptosis via the ATF4‒SLC7A11 pathway.

To test our hypothesis, we first demonstrated the relationships among METTL9, SLC39A7, and the ATF4/SLC7A11 pathway. Western blotting and quantification revealed that METTL9 expression was positively correlated with ATF4/SLC7A11 expression (Fig. 6E). SLC39A7 wt expression was negatively correlated with ATF4/SLC7A11 expression, and SLC39A7 mut inhibited the negative regulatory effect of SLC39A7 wt on ATF4/SLC7A11 (Fig. 6F). We then designed four experimental combinations: SLC39A7 wt + Vector, SLC39A7 wt + METTL9, SLC39A7 mut, and SLC39A7 mut + METTL9. Western blotting and quantification revealed that METTL9 overexpression promoted PERK phosphorylation and increased ATF4/SLC7A11 expression, but this effect was not detected in MSCs expressing the SLC39A7 mutant (Fig. 6G).

Since SLC7A11 is the key transporter that is responsible for extracellular cystine uptake and since extracellular cystine is converted to cysteine inside the cell and used for glutathione (GSH) synthesis, we further investigated whether the METTL9-SLC39A7 axis regulates intracellular cysteine and GSH levels. The results revealed that METTL9 overexpression increased the intracellular GSH and cysteine levels, but this effect was not detected in cells expressing the SLC39A7 mutant (Fig. 6H, I). In conclusion, our findings suggest that the METTL9‒SLC39A7 axis can inhibit ferroptosis by increasing the intracellular cysteine and GSH levels via the ATF4/SLC7A11 pathway.

### Overexpression of METTL9 alleviates osteoporosis in mice

**Fig. 7:**
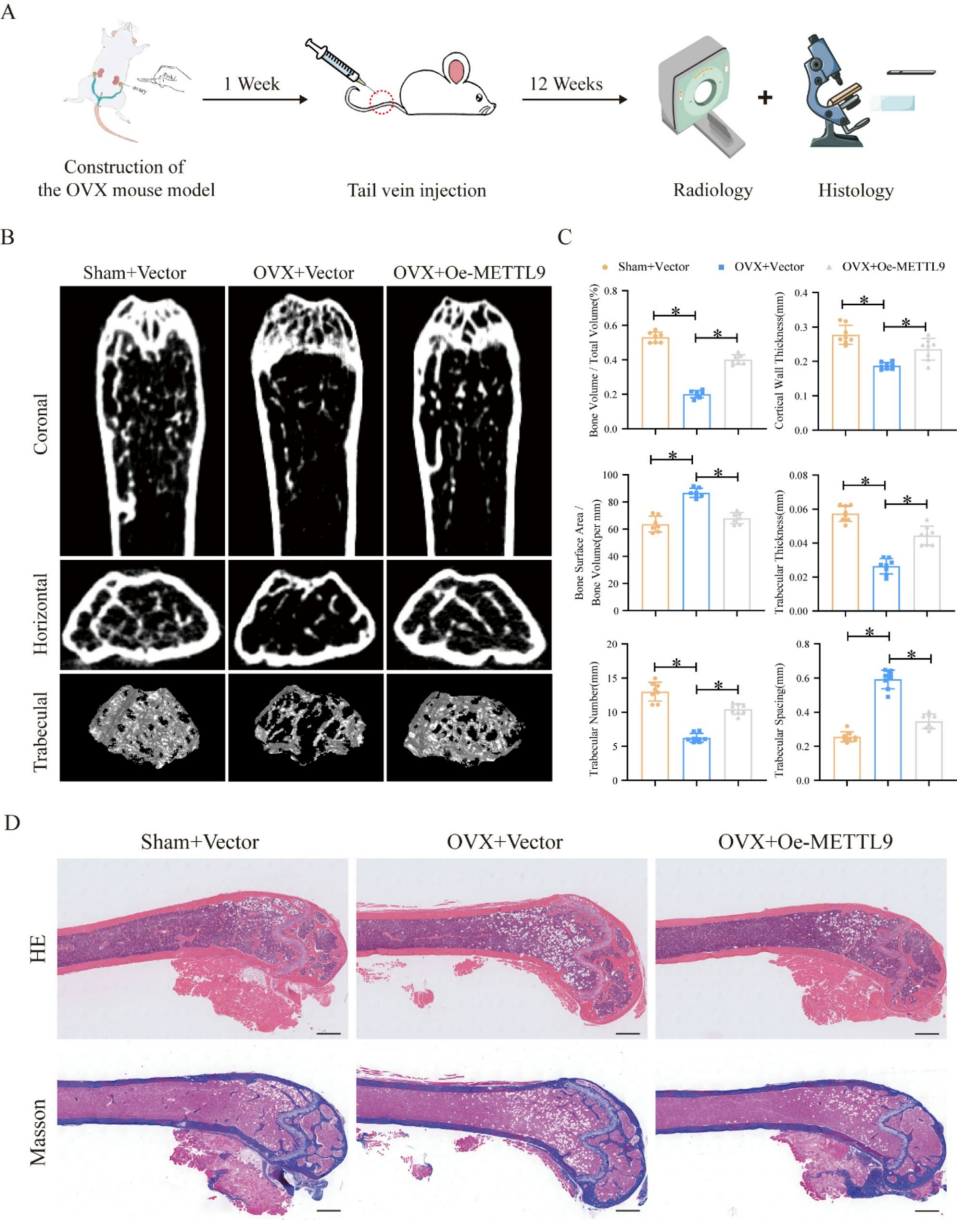
Overexpression of METTL9 alleviates osteoporosis in mice. **A** Schematic diagram showing the establishment of the OVX mouse model, rAAV9-METTL9 tail vein injection, and bone tissue section analysis. **B** Micro-CT images showing coronal, transverse, and trabecular bone sections of femurs from normal mice, OVX-treated mice, and METTL9-overexpressing mice. **C** Calculations of bone morphological parameters in the region of interest (ROI). **D** H&E staining and Masson staining demonstrating changes in bone marrow adipose tissue and bone mass in the mice. The scale bar in the images represents 500 μm. Data are presented as mean ± SD of eight biologically independent replicates, *p < 0.05

Osteoporosis is a common age-related disease, and MSCs play key roles in the initiation, progression, and treatment of osteoporosis. To further verify whether METTL9 could be used as a therapeutic target for osteoporosis in vivo, we established an OVX-induced model of osteoporosis in mice and injected rAAV9-METTL9 into the mice via the tail vein 1 week later. Twelve weeks later, the femurs were collected for micro-CT scanning and tissue section analysis (Fig. 7A).

The micro-CT results revealed that the trabecular bone count in the METTL9 overexpressing group was significantly greater than that in the OVX group (Fig. 7B). Additionally, we performed quantitative analysis of several trabecular bone parameters (bone surface area/bone volume, trabecular volume/total marrow cavity volume, trabecular thickness, trabecular number, trabecular spacing, and cortical thickness), and the results were consistent with the CT data (Fig. 7C). H&E and Masson’s trichrome staining of paraffin sections further demonstrated a significant increase in trabecular bone quantity and a marked reduction in the number of lipid droplets in the bone marrow in the METTL9 overexpressing group (Fig. 7D).

In conclusion, these results suggest that increasing METTL9 levels in osteoporotic mice can effectively mitigate osteoporosis progression, making METTL9 a potential target for the diagnosis and treatment of osteoporosis.

## Discussion

Osteoporosis is a common metabolic disease that affects more than 200 million people worldwide, and it is associated with high health care costs (Reginster et al. 2006). An imbalance between the adipogenic and osteogenic differentiation of MSCs plays a critical role in the pathogenesis of osteoporosis, but the detailed mechanisms that affect MSC lineage allocation remain unclear. Previous studies have shown that a shift in MSC differentiation toward the adipocyte lineage leads to progressive bone loss and increased bone marrow adipose tissue formation, thereby promoting osteoporosis development (Ambrosi et al. 2017; Fazeli et al. 2013). Therefore, investigating the underlying molecular mechanisms that affect the balance of adipogenic and osteogenic differentiation of MSCs is essential for identifying potential therapeutic targets for osteoporosis. Our findings indicate that METTL9 expression is downregulated in osteoporosis, and we further demonstrated that METTL9 negatively regulates MSC adipogenic differentiation both in vitro and in vivo. Furthermore, METTL9 overexpression in OVX-treated mice markedly reduced bone loss. Therefore, our findings suggest that METTL9 plays a crucial role in inhibiting bone marrow adipose tissue growth and maintaining the balance between adipogenic and osteogenic differentiation.

However, the mechanism by which METTL9 inhibits the adipogenic differentiation of MSCs remains the central question of this study. Erna Davydova et al. (2021) discovered that METTL9 catalyzes the N1-methylation of protein substrates that contain the “x-His-x-His” motif (where “x” represents a small amino acid, and “H” represents histidine), and they identified six potential substrates that can be methylated by METTL9 both in vitro and in vivo. Through bioinformatics analysis and adipogenic functional validation, we identified SLC39A7 as the most likely downstream target of METTL9 among the six potential substrates. Our research revealed that after METTL9 knockdown in MSCs, the methylation of SLC39A7 was significantly reduced. Additionally, further adipogenic functional experiments demonstrated that the adipogenic differentiation of MSCs was weakened after SLC39A7 knockdown. Furthermore, we confirmed that SLC39A7 expression was significantly upregulated in osteoporosis, which was confirmed at the gene, protein, and tissue levels. SLC39A7 is a zinc transporter that is primarily responsible for maintaining zinc homeostasis within the ER. Specifically, John Adulcikas et al. (2018) reported that the N-terminal region of SLC39A7 is rich in histidine residues and contains many “xHxH” motifs, which facilitate the ability of SLC39A7 to bind to and transport zinc. Research has shown that zinc transporters participate in zinc storage and release, and abnormalities in zinc transporter function are associated with the disruption of bone homeostasis, which may lead to bone diseases in humans (Huang et al. 2020). Numerous studies have revealed the role of zinc transporters in osteoporosis. For example, Zhihui Tang et al. (2006) reported that ZiP1 mediates the influx of zinc during MSC differentiation into osteoblasts and that ZiP1 overexpression increases the expression of osteogenic markers. In addition, Yang Liu et al. (2013) reported that ZnT7 regulates the osteogenic differentiation of MSCs by regulating the Wnt and ERK signaling pathways. In summary, zinc transporter dysfunction may lead to an imbalance between the adipogenic and osteogenic differentiation of MSCs. In our study, we substituted the histidine residues at positions 45 and 49 in the N-terminal region of SLC39A7 with alanine. Our experimental results revealed that the ability of the SLC39A7 mutant to transport zinc ions was significantly reduced, and its capacity to induce adipogenic differentiation was notably decreased. Therefore, the His45 and His49 sites of SLC39A7 are crucial for maintaining the zinc transport ability of this protein and promoting MSC adipogenic differentiation. On the basis of these results, we conclude that METTL9 functions upstream of SLC39A7, inhibiting its ability to transport zinc by methylating His45 and His49, thereby suppressing MSC adipogenic differentiation. Overall, our study is the first to report that METTL9 negatively regulates the adipogenic differentiation of MSCs via the methylation of SLC39A7 at the His45 and His49 residues.

The specific mechanism by which METTL9 inhibits the adipogenic differentiation of MSCs via the methylation of SLC39A7 is another key question. Recent studies have shown that SLC39A7 is a key regulator of ferroptosis and that both genetic knockout and chemical inhibition of SLC39A7 significantly inhibit ferroptosis (Chen et al. 2021). Fangfang Bi et al. (2023) reported that METTL9 is a novel regulator of ferroptosis. In our study, we observed that changes in METTL9 and SLC39A7 expression were both correlated with the levels of ROS in cells and mitochondria. METTL9 mediated methylation of SLC39A7 reduced lipid peroxidation levels, suggesting that METTL9 may regulate ferroptosis via the methylation of SLC39A7. Ferroptosis is a form of nonapoptotic cell death that is characterized by the accumulation of lipid peroxides, and plays a role in the pathogenesis of various diseases, including neurodegenerative diseases, ischemic injury, and cancer (Fang et al. 2019; Liang et al. 2019; Maher et al. 2020). In recent years, the anti-osteoporotic role of ferroptosis has been revealed, and thus, ferroptosis has emerged as a new target for the treatment of osteoporosis. Yiqi Yang et al. (2022) reported that the interaction between NRF2 and c-JUN induces ferroptosis by upregulating HO-1 and that targeting the ferroptosis of osteoblast can effectively prevent bone loss. Zengxin Jiang et al. (2024) also reported a novel therapeutic strategy to prevent and treat postmenopausal osteoporosis by inhibiting the ferroptosis of osteocytes via the NRF2/Dnmt3a/RANKL axis. In our study, the ferroptosis inhibitor FEO reduced the adipogenic differentiation of MSCs, suggesting that inhibiting ferroptosis not only suppresses the progression of osteoporosis by increasing the activity of osteoblasts and osteocytes but also influences the adipogenic-osteogenic differentiation direction of MSCs.

**Fig. 8:**
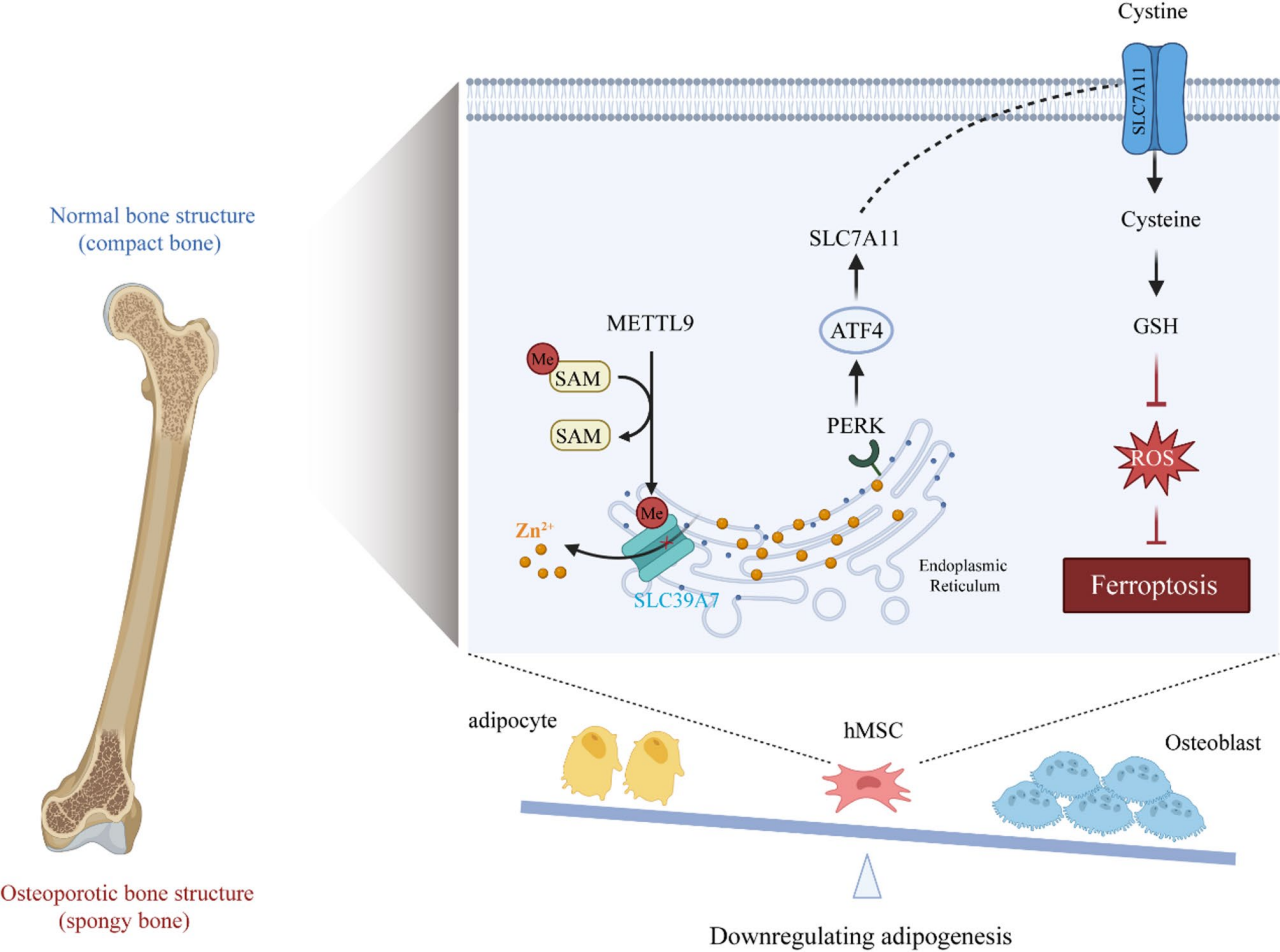
A schematic diagram of the mechanism by which METTL9 affects osteogenic differentiation. The diagram shows that the METTL9/SLC39A7 axis inhibits ferroptosis by regulating the expression of ATF4/SLC7A11, thereby suppressing the adipogenic differentiation of MSCs. Created with BioRender.com

Our subsequent experimental results revealed that Si-SLC39A7 led to the activation of the PERK‒ATF4 pathway during ER stress. ATF4 induces the transcription of various stress-dependent genes, including SLC7A11 (Chen et al. 2017; Marciniak et al. 2022). SLC7A11 is a cystine transporter, and it plays a key role in glutathione synthesis and maintaining the cellular redox balance as well as a crucial role in inhibiting ferroptosis (Chen et al. 2024; He et al. 2023). More importantly, ameliorating oxidative stress favors the differentiation of MSCs toward osteoblasts rather than adipocytes (Atashi et al. 2015). Therefore, we conclude that METTL9 activates the ATF4/SLC7A11 axis by methylating SLC39A7. As a key inhibitor of ferroptosis, SLC7A11 controls the transport of extracellular cystine and thus the production of glutathione (GSH), subsequently scavenging intracellular ROS and inhibiting MSC adipogenic differentiation (Fig. 8).

Elucidating the pathogenesis of osteoporosis and identifying effective treatments are key challenges in this field. Currently, bisphosphonates, denosumab, and teriparatide are the primary agents that are used to treat osteoporosis (Khosla et al. 2017). However, despite their efficacy in preventing or alleviating osteoporosis, their side effects, patient compliance, and costs cannot be overlooked. For example, long-term use of bisphosphonates may lead to osteonecrosis of the jaw, and patients with renal insufficiency are strictly prohibited from using them (Kennel et al. n.d.). The discontinuation of denosumab leads to a severe rebound effect, which is characterized by decreased bone density and a significantly increased risk of vertebral fractures (Elbers et al. 2021). Teriparatide and other drugs that promote bone formation have limited use durations, are expensive, and require good patient compliance for daily subcutaneous injections (Blick et al. 2008). Therefore, further development of new drugs that avoid these side effects and have long-term effects on bone anabolism is necessary. To investigate whether METTL9 could be used as a therapeutic target for osteoporosis, we injected an adenovirus overexpressing METTL9 into a mouse model of osteoporosis. The results showed that METTL9 overexpression effectively inhibited adipose tissue growth and reduced bone loss in the mouse osteoporosis model. These findings indicate that METTL9 is a key target in the progression of osteoporosis, and it may be used as an alternative therapeutic target.

In conclusion, METTL9 plays a crucial role in regulating the balance between the adipogenic and osteogenic differentiation of MSCs in osteoporosis, and thus, it is a potential target for the diagnosis and treatment of osteoporosis. However, our study has several limitations. For example, clinical trials that validate whether METTL9 can be used as a clinical therapeutic target are lacking. Additionally, the upstream regulatory mechanisms that lead to reduced METTL9 expression in individuals with osteoporosis remain unclear. Future research is needed to address these issues.

## Supplementary Information

Supplementary Material 1.

Supplementary Material 2.

Supplementary Material 3.

## Data Availability

No datasets were generated or analysed during the current study.
