# Supplementary material for: METTL9 mediated N1-Histidine methylation of SLC39A7 confers ferroptosis resistance and inhibits adipogenic differentiation in mesenchymal stem cells
Source: Mol Med. 2025 May 26;31:206. doi: 10.1186/s10020-025-01271-w (PMC12105315; doi:10.1186/s10020-025-01271-w)
Supplement: Supplementary file 1 — Supplementary Material 1. [file 10020_2025_1271_MOESM1_ESM.doc]

| **Supplemental Table S1. Primer sequences for real-time PCR** | | | | |
| --- | --- | --- | --- | --- |
| Primer sequence | | | | |
| METTL9 for human | | Forward |  | 5’-CCTGTACGTGAACATGACTAGCG-3' |
|  |  | Reverse |  | 5’-AAGACAGCCTGGAGTGATTCGC-3' |
| SLC39A7 for human | | Forward |  | 5’-TGAGACCTCAGAACGCTGAAG-3' |
|  |  | Reverse |  | 5’-CTCGAAAGGAAGCCCCAATGG-3' |
| PPAR-γ for human | | Forward |  | 5'-GGGATCAGCTCCGTGGATCT-3' |
|  |  | Reverse |  | 5'-TGCACTTTGGTACTCTTGAAGTT-3' |
| C/EBPα for human | | Forward |  | 5'-TATAGGCTGGGCTTCCCCTT-3' |
|  |  | Reverse |  | 5'-AgCTTTCTGGTGTGACTCGG-3' |
| FABP4 for human | | Forward |  | 5’-ACTGGGCCAGGAATTTGACG-3’ |
|  |  | Reverse |  | 5’-CTCGTGGAAGTGACGCCTT-3’ |
| ATF6 for human | | Forward |  | 5'-AGCAGCACCCAAGACTCAAAC-3' |
|  |  | Reverse |  | 5'-GCATAAGCGTTGGTACTGTCTGA-3' |
| PERK for human | | Forward |  | 5’-ACGATGAGACAGAGTTGCGAC-3’ |
|  |  | Reverse |  | 5’-ATCCAAGGCAGCAATTCTCCC-3’ |
| IRE1 for human |  | Forward |  | 5’-AGAGAAGCAGCAGACTTTGTC-3' |
|  |  | Reverse |  | 5’-GTTTTGGTGTCGTACATGGTGA-3' |
| SLC7A11 for human |  | Forward |  | 5’-TCCTGCTTTGGCTCCATGAACG-3' |
|  |  | Reverse |  | 5’-AGAGGAGTGTGCTTGCGGACAT-3' |
| HMOX1 for human |  | Forward |  | 5’-AAGACTGCGTTCCTGCTCAAC-3' |
|  |  | Reverse |  | 5’-AAAGCCCTACAGCAACTGTCG-3' |
| SLC3A2 for human |  | Forward |  | 5’-TGAATGAGTTAGAGCCCGAGA-3' |
|  |  | Reverse |  | 5’-GTCTTCCGCCACCTTGATCTT-3' |
| SLC39A14 for human |  | Forward |  | 5’-TCCTGGCTGGCAGTCACTTCTC-3' |
|  |  | Reverse |  | 5’-TTCTCATCCTCCTGGCACACCTC-3' |
| GAPDH for human |  | Forward |  | 5'-GGAGCGAGATCCCTCCAAAAT-3' |
|  |  | Reverse |  | 5'-GGCTGTTGTCATACTTCTCATGG-3' |

Abbreviations: *METTL9*, methyltransferase 9, His-X-His N1(pi)-histidine; *SLC39A7*, solute carrier family 39 member 7; *PPAR-γ*, peroxisome proliferator-activated receptor gamma; *C/EBP-α*, CCAAT/enhancer binding protein alpha; *FABP4*, fatty acid binding protein 4; *ATF6*, activating transcription factor 6; *PERK*, eukaryotic translation initiation factor 2 alpha kinase 3; *IRE1*, bifunctional endoribonuclease/protein kinase IRE1; *SLC7A11*, solute carrier family 7 member 11; *HMOX1*, heme oxygenase 1; *SLC3A2*, solute carrier family 3 member 2; *SLC39A14*, solute carrier family 39 member 14. *GAPDH*, glyceraldehyde-3-phosphate dehydrogenase.

| **Supplemental Table S2. siRNA sequences for METTL9** | | | | | |
| --- | --- | --- | --- | --- | --- |
| siRNA sequence | | | | | |
| METTL9-siRNA1 | |  | sense |  | 5′-GGAGAACCACCAGUGGUAUTT-3′ |
|  |  |  | antisense |  | 5'-AUACCACUGGUGGUUCUCCTT-3′ |
| METTL9-siRNA2 | |  | sense |  | 5′-GGGCUGGCUAUUUAUCCAATT-3′ |
|  |  |  | antisense |  | 5′-UUGGAUAAAUAGCCAGCCCTT-3′ |
| METTL9-siRNA3 | |  | sense |  | 5′-GGUUCCAGUAUGAUGUCAUTT-3′ |
|  |  |  | antisense |  | 5′-AUGACAUCAUACUGGAACCTT-3′ |
| METTL9-siRNA4 |  |  | sense |  | 5′-GCGACAUGUAUAAUGACUATT-3′ |
|  |  |  | antisense |  | 5′-UAGUCAUUAUACAUGUCGCTT-3′ |

| **Supplemental Table S3. siRNA sequences for SLC39A7** | | | | | |
| --- | --- | --- | --- | --- | --- |
| siRNA sequence | | | | | |
| SLC39A7-siRNA1 | |  | sense |  | 5′-GGCACUCACAUGAAGAUUUTT-3′ |
|  |  |  | antisense |  | 5'-AAAUCUUCAUGUGAGUGCCTT-3′ |
| SLC39A7-siRNA2 | |  | sense |  | 5′-GGAUUUACACCAUGGCCAUTT-3′ |
|  |  |  | antisense |  | 5′-AUGGCCAUGGUGUAAAUCCTT-3′ |
| SLC39A7-siRNA3 | |  | sense |  | 5′-GGAGAAAUUUGUGAGACAUTT-3′ |
|  |  |  | antisense |  | 5′-AUGUCUCACAAAUUUCUCCTT-3′ |
| SLC39A7-siRNA4 |  |  | sense |  | 5′-GGAGGCAUCACCAUUGCAATT-3′ |
|  |  |  | antisense |  | 5′-UUGCAAUGGUGAUGCCUCCTT-3′ |

| **Supplemental Table S4. siRNA sequences for DNAJB12** | | | | | |
| --- | --- | --- | --- | --- | --- |
| siRNA sequence | | | | | |
| DNAJB12-siRNA1 | |  | sense |  | 5′- GGAAUCCAACAAGGAUGAATT-3′ |
|  |  |  | antisense |  | 5'- UUCAUCCUUGUUGGAUUCCTT-3′ |
| DNAJB12-siRNA2 | |  | sense |  | 5′- CCAUGCCACCCACAGGAAATT-3′ |
|  |  |  | antisense |  | 5′- UUUCCUGUGGGUGGCAUGGTT-3′ |
| DNAJB12-siRNA3 | |  | sense |  | 5′- GCGGCUUCCCUUCUAGUAATT-3′ |
|  |  |  | antisense |  | 5′- UUACUAGAAGGGAAGCCGCTT-3′ |
| DNAJB12-siRNA4 |  |  | sense |  | 5′- GCGCUAUACCUACCAGCAATT-3′ |
|  |  |  | antisense |  | 5′- UUGCUGGUAGGUAUAGCGCTT-3′ |

| **Supplemental Table S5. siRNA sequences for CCNT2** | | | | | |
| --- | --- | --- | --- | --- | --- |
| siRNA sequence | | | | | |
| CCNT2-siRNA1 | |  | sense |  | 5′- GGAGCUUCUUCUCGCUGGUUCUUUATT-3′ |
|  |  |  | antisense |  | 5′- UAAAGAACCAGCGAGAAGAAGCUCCTT-3′ |
| DNAJB12-siRNA2 | |  | sense |  | 5′- GCCACUGCUGGAUACUAAAUGUGAUTT-3′ |
|  |  |  | antisense |  | 5′- AUCACAUUUAGUAUCCAGCAGUGGCTT-3′ |
| DNAJB12-siRNA3 | |  | sense |  | 5′- CAGUUAGUAAGAGCAAGCAAGGAUUTT-3′ |
|  |  |  | antisense |  | 5′- AAUCCUUGCUUGCUCUUACUAACUGTT-3′ |
| DNAJB12-siRNA4 |  |  | sense |  | 5′- CAGGGACCUUCUAUAUCACUGCAUUTT-3′ |
|  |  |  | antisense |  | 5′- AAUGCAGUGAUAUAGAAGGUCCCUGTT-3′ |

| **Supplemental Table S6. siRNA sequences for Si-Ctrl** | | | | | |
| --- | --- | --- | --- | --- | --- |
| siRNA sequence | | | | | |
| Control-siRNA | |  | sense |  | 5′- UUCUCCGAACGUGUCACGUTT -3′ |
|  |  |  | antisense |  | 5′- ACGUGACACGUUCGGAGAATT -3′ |

**Supplementary Table S****7. Characteristics of the study subjects**

|  | Normal control | Osteoporosis Patients |
| --- | --- | --- |
| Number | 6 | 6 |
| Age (years) | 37.64±12.51 | 76±7.49 |
| Sex | Female | Female |
| Hight (cm) | 158.26±6.34 | 157.47±8.09 |
| Weight (kg) | 54.46±6.46 | 58.52±4.63 |
| BMI (kg/m2) | 21.65±0.85 | 23.62±0.56 |
| Age of menarche (years) | 13.41±1.18 | 13.25±1.28 |
| Age of menopause (years) | / | 56.34±3.62 |
| Lumbar spine BMD (g/cm2) | 1.36±0.42 | 0.72±0.33 |
| Lumbar spine T score | 0.49±0.19 | -2.59±0.49 |
| Total hip BMD (g/cm2) | 1.38±0.42 | 0.51±0.24 |
| Total hip T score | 0.47±0.23 | -1.25±0.38 |

Data are shown as the mean ± SD, n=6 in each group. P values for all variables are the result of independent t tests between the control and osteoporosis groups, BMI, body mass index; BMD, bone mineral density.
