# Supplementary material for: METTL9 mediated N1-Histidine methylation of SLC39A7 confers ferroptosis resistance and inhibits adipogenic differentiation in mesenchymal stem cells
Source: Mol Med. 2025 May 26;31:206. doi: 10.1186/s10020-025-01271-w (PMC12105315; doi:10.1186/s10020-025-01271-w)
Supplement: Supplementary file 2 — Supplementary Material 2. [file 10020_2025_1271_MOESM2_ESM.doc]

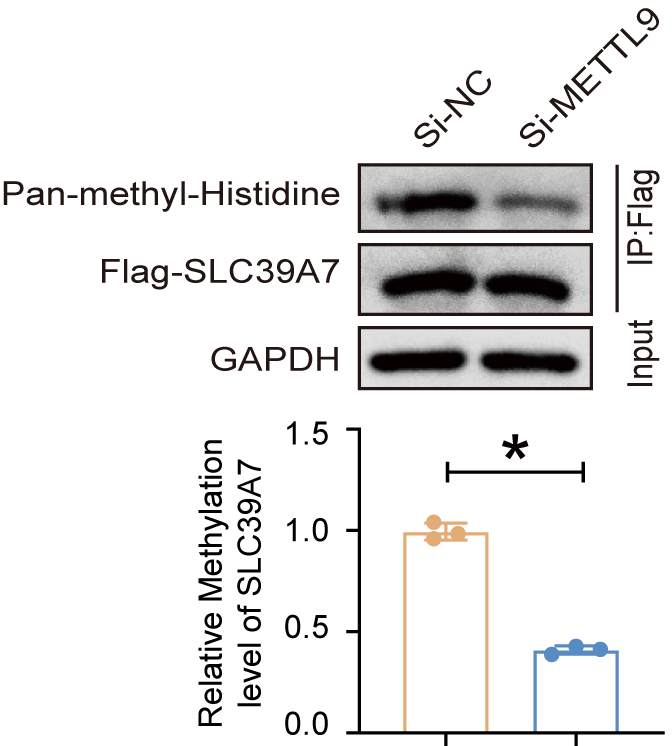


***Figure. 4-figure supplement 1*** Immunoblotting was used to measure the methylation levels of Si-NC and Si-METTL9. All the data are presented as the mean ± SD, n = 3 per group, *p < 0.05. All experiments were independently repeated three times.


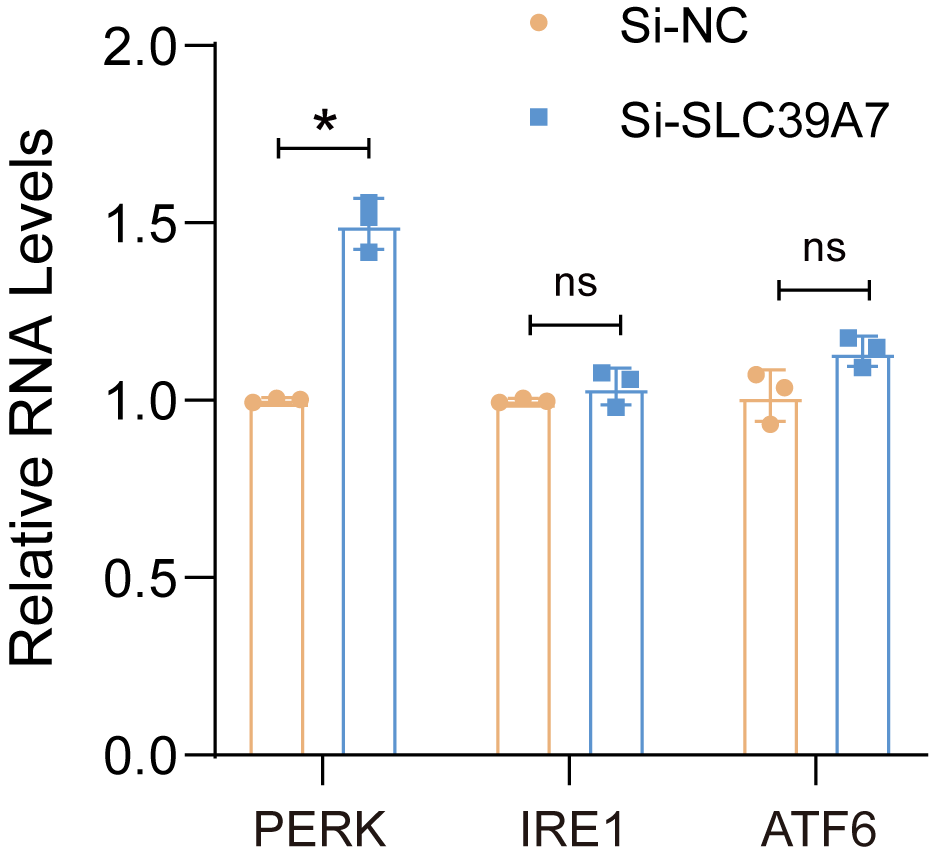


***Figure. 6-figure supplement 1*** RT‒qPCR analysis of the mRNA expression of PERK, IRE1 and ATF6 after Si-SLC39A7 treatment. All the data are presented as the mean ± SD, n = 3 per group, *p < 0.05. All experiments were independently repeated three times.
