# Supplementary material for: METTL9 mediated N1-Histidine methylation of SLC39A7 confers ferroptosis resistance and inhibits adipogenic differentiation in mesenchymal stem cells
Source: Mol Med. 2025 May 26;31:206. doi: 10.1186/s10020-025-01271-w (PMC12105315; doi:10.1186/s10020-025-01271-w)

Fig.1H （in paper）

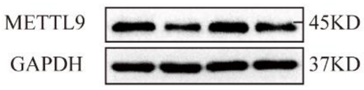

Raw data

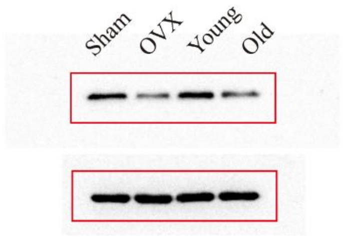

Fig.2B （in paper）

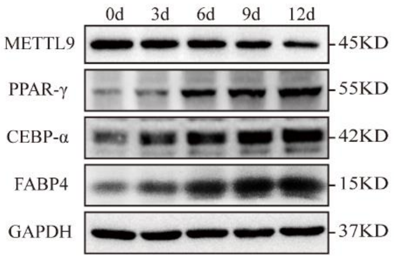

Raw data

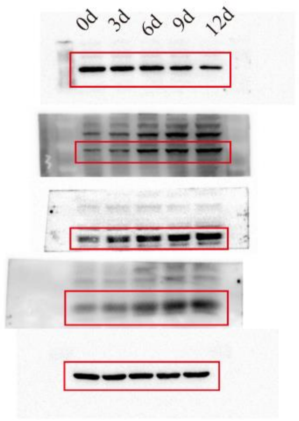

Fig.2G （in paper）

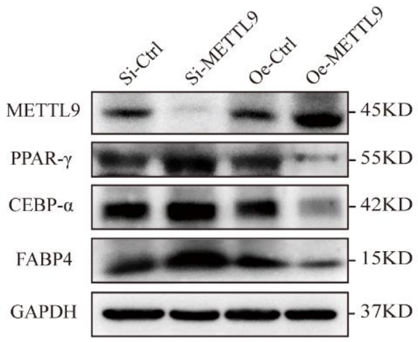

Raw data

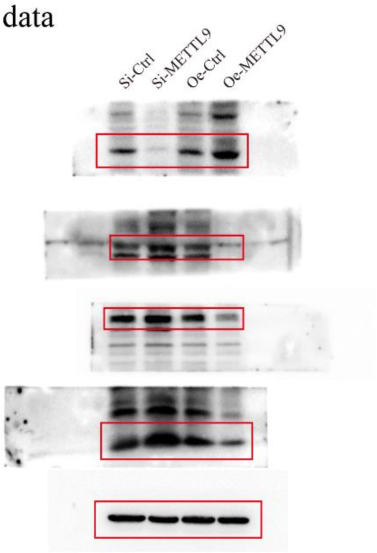

Fig.3C （in paper）

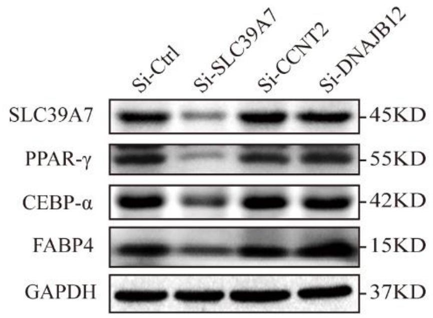

Raw data

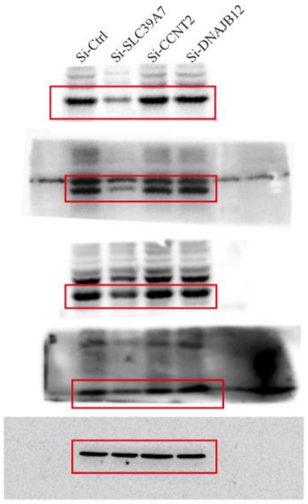

Fig.3H （in paper）

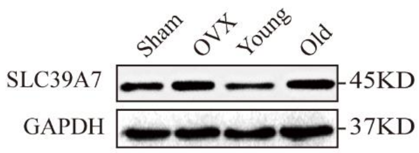

Raw data

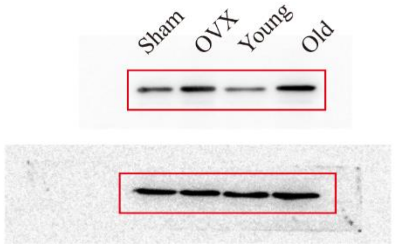

Fig.4B （in paper）

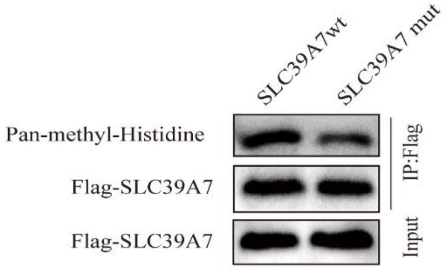

Raw data

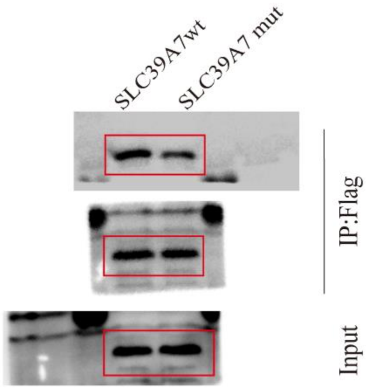

Fig.4D （in paper）

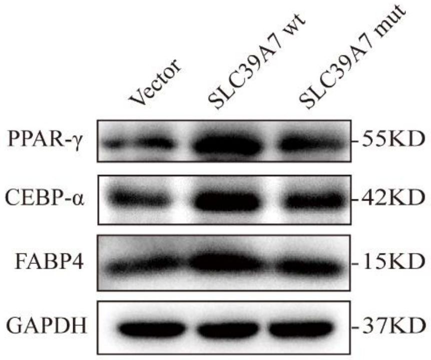

Raw data

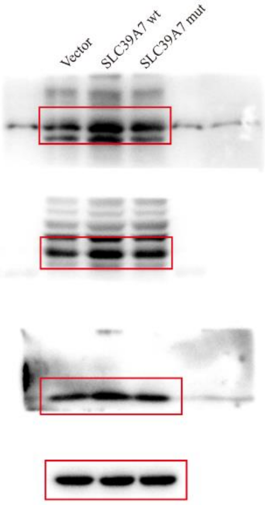

Fig.4G （in paper）

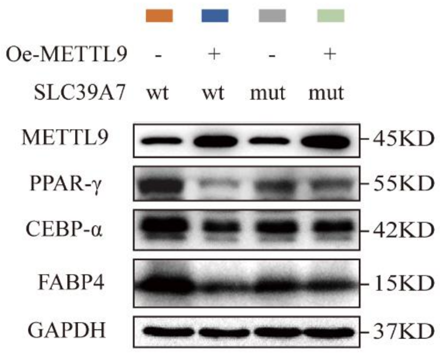

Raw data

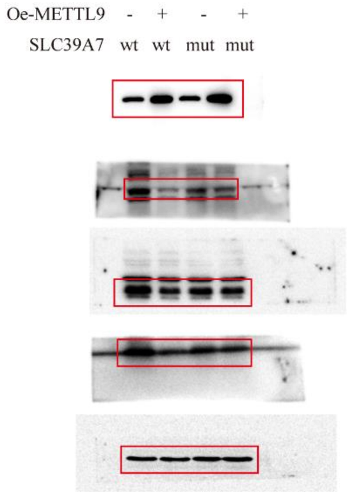

Fig.5H（in paper）

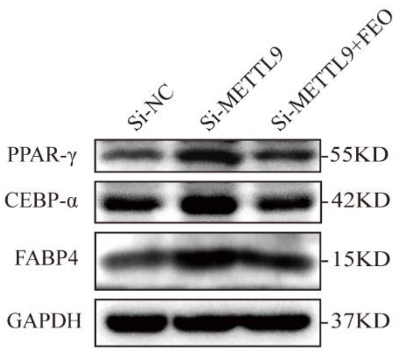

Raw data

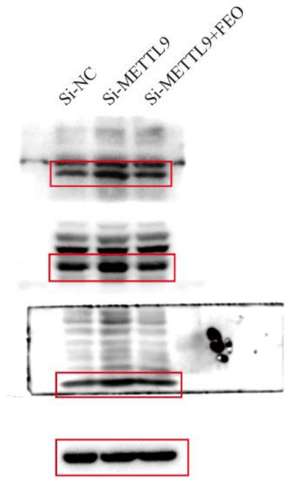

Fig.5J（in paper）

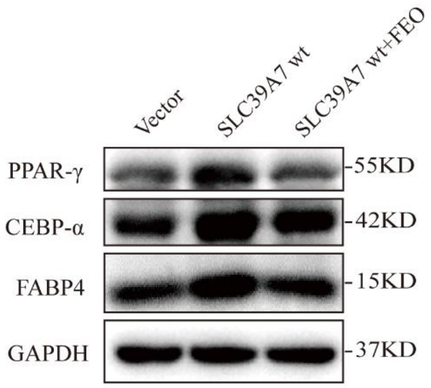

Raw data

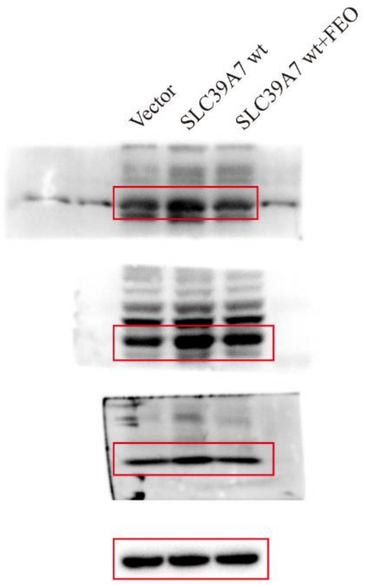

Fig.6C （in paper）

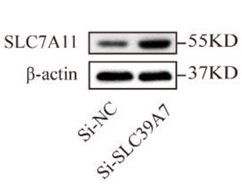

Raw data

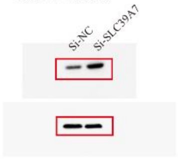

Fig.6D （in paper）

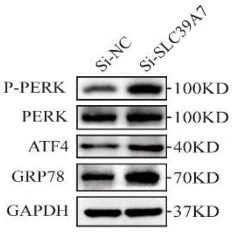

Raw data

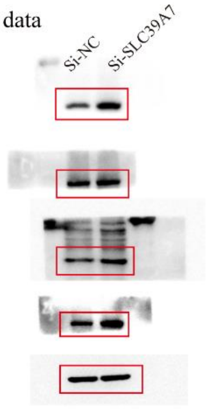

Fig.6E （in paper）

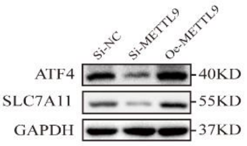

Raw data

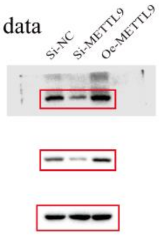

Fig.6F （in paper）

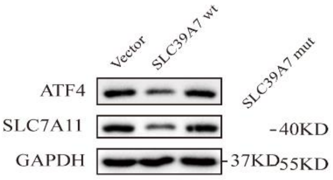

Raw data

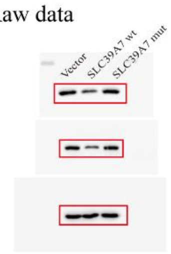

Fig.6G （in paper）

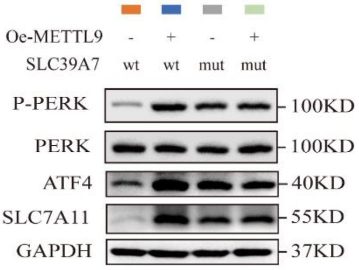

Raw data

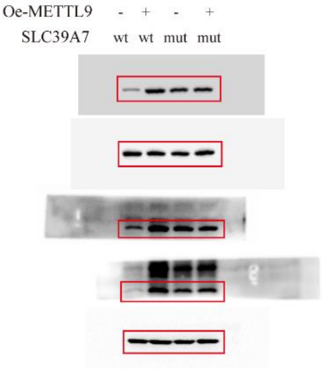

Supplement: Supplementary file 3 — Supplementary Material 3. [file 10020_2025_1271_MOESM3_ESM.pdf]
